# Supplementary material for: The effects of protein supplementation, fumagillin treatment, and colony management on the productivity and long-term survival of honey bee (Apis mellifera) colonies
Source: PLoS One. 2024 Mar 15;19(3):e0288953. doi: 10.1371/journal.pone.0288953 (PMC10942092; doi:10.1371/journal.pone.0288953)
Supplement: S5 File — HTML file showing the output of the statistical analysis. (HTML) [file pone.0288953.s005.html]

Supplement 5: Statistical Analysis


# Supplement 5: Statistical Analysis

Original analysis with:

RStudio build 576 “Spotted Wakerobin”

R version 4.2.2 (2022-10-31 ucrt) “Innocent and Trusting” Platform:
x86\_64-w64-mingw32/x64 (64-bit) Running under: Windows 10 x64 (build
19044)

Packages:

car\_3.1-1 carData\_3.0-5 survival\_3.4-0 emmeans\_1.8.2 nlme\_3.1-160
pander\_0.6.5 dplyr\_1.0.10

See bottom of document for current settings.

# **1. Colony Survival**

Colony survival was analyzed as a Cox proportional hazards model.
Region was a stratified factor because a prior version of the model
produced results indicating that hazards were not proportional among
regions. For colonies in the fumagillin treated group, the pre-treatment
period and post treatment period were distinguished in the analyzed
dataset using the tmerge function. The cluster function and robust
standard errors were used to account for relatedness among colonies
split from a single parent. The final model below was reduced from the
model showing all interactions of the three factors (Region, Patties,
Fumagillin) by successively removing non-significant higher order
interactions.

#

# 

**Model Code in R:**

cphfr <- coxph(Surv(tstart, tstop, death) ~ strata(Region) \*
Patties + Fume, newBHPsurv, cluster=ColonyGroup)

# 

**Analysis of Variance**

skipping term strata(Region)

Analysis of Deviance Table (Type II tests)


|  | Df | Chisq | Pr(>Chisq) |
| --- | --- | --- | --- |
| **Patties** | 1 | 5.325 | 0.02102 |
| **Fume** | 1 | 1.284 | 0.2572 |
| **strata(Region):Patties** | 2 | 6.493 | 0.0389 |

# 

**Effect Estimates**

Fitting Proportional Hazards Regression Model: Surv(tstart,
tstop, death) ~ strata(Region) \* Patties + Fume


|  | coef | exp(coef) | robust se | z | p |
| --- | --- | --- | --- | --- | --- |
| **PattiesYes** | 0.02028 | 1.02 | 0.352 | 0.05761 | 1 |
| **Fume** | -0.1687 | 0.8447 | 0.1489 | -1.133 | 0.3 |
| **strata(Region)Northern Alberta:PattiesYes** | 0.8762 | 2.402 | 0.4416 | 1.984 | 0.05 |
| **strata(Region)Prince Edward Island:PattiesYes** | 0.1105 | 1.117 | 0.3959 | 0.2791 | 0.8 |

Likelihood ratio test=12.73 on 4 df, p=0.01266398 n= 531, number of
events= 194

**Hazard Ratios**

p values are shown without adjustment for multiple comparisons. For
the within-region effects, the Bonferroni-adjusted threshold for
evaluating significance would be p=0.05/3 = 0.0167.

| Patties effect | Region | ratio | SE | df | asymp.LCL | asymp.UCL | null | z.ratio | p.value |
| --- | --- | --- | --- | --- | --- | --- | --- | --- | --- |
| Yes / No | Southern Alberta | 1.02 | 0.3592 | Inf | 0.5119 | 2.034 | 1 | 0.05761 | 0.9541 |
| Yes / No | Northern Alberta | 2.451 | 0.6536 | Inf | 1.453 | 4.134 | 1 | 3.362 | 0.0007743 |
| Yes / No | Prince Edward Island | 1.14 | 0.2067 | Inf | 0.7988 | 1.626 | 1 | 0.7211 | 0.4709 |

| Fumagillin effect | ratio | SE | df | asymp.LCL | asymp.UCL | null | z.ratio | p.value |
| --- | --- | --- | --- | --- | --- | --- | --- | --- |
| Fume1 / Fume0 | 0.8447 | 0.1258 | Inf | 0.6309 | 1.131 | 1 | -1.133 | 0.2572 |

# 

**Proportional Hazards Assumption Test**

|  | chisq | df | p |
| --- | --- | --- | --- |
| **Patties** | 1.849 | 1 | 0.1739 |
| **Fume** | 0.2036 | 1 | 0.6518 |
| **strata(Region):Patties** | 6.151 | 2 | 0.04617 |
| **GLOBAL** | 6.387 | 4 | 0.1721 |

# **2. Queen Survival - Successful Supercedures Only (Both Natural and Manual)**

Queen survival was analyzed as a Cox proportional hazards model.
Region was a stratified factor because a prior version of the model
produced results indicating that hazards were not proportional among
regions. For colonies in the fumagillin treated group, the pre-treatment
period and post treatment period were distinguished in the analyzed
dataset using the tmerge function. The cluster function and robust
standard errors were used to account for relatedness among colonies
split from a single parent. Multiple queen events per colony were
possible and distinguished in the dataset using the tmerge function.
Only queen deaths that were not associated with a colony death were
included in this model. The final model below was reduced from the model
showing all interactions of the three factors (Region, Patties,
Fumagillin) by successively removing non-significant higher order
interactions.

**Model Code in R:**

BHPtr<-coxph(Surv(tstart, tstop, Queen) ~ strata(Region) + Patties
+ Fume, Qsurv, cluster=ColonyGroup)

**Analysis of Variance**

skipping term strata(Region)

Analysis of Deviance Table (Type II tests)


|  | Df | Chisq | Pr(>Chisq) |
| --- | --- | --- | --- |
| **Patties** | 1 | 0.675 | 0.4113 |
| **Fume** | 1 | 0.1721 | 0.6783 |

**Effect Estimates**

Fitting Proportional Hazards Regression Model: Surv(tstart,
tstop, Queen) ~ strata(Region) + Patties + Fume


|  | coef | exp(coef) | robust se | z | p |
| --- | --- | --- | --- | --- | --- |
| **PattiesYes** | 0.1063 | 1.112 | 0.1294 | 0.8216 | 0.4 |
| **Fume** | -0.07602 | 0.9268 | 0.1833 | -0.4148 | 0.7 |

Likelihood ratio test=0.64 on 2 df, p=0.7263979 n= 695, number of
events= 181

**Hazard Ratios**

| Patties effect | ratio | SE | df | asymp.LCL | asymp.UCL | null | z.ratio | p.value |
| --- | --- | --- | --- | --- | --- | --- | --- | --- |
| Yes / No | 1.112 | 0.1439 | Inf | 0.863 | 1.433 | 1 | 0.8216 | 0.4113 |

| Fumagillin effect | ratio | SE | df | asymp.LCL | asymp.UCL | null | z.ratio | p.value |
| --- | --- | --- | --- | --- | --- | --- | --- | --- |
| Fume1 / Fume0 | 0.9268 | 0.1698 | Inf | 0.6471 | 1.327 | 1 | -0.4148 | 0.6783 |

**Test of Hazard Proportionality**

|  | chisq | df | p |
| --- | --- | --- | --- |
| **Patties** | 0.19 | 1 | 0.6629 |
| **Fume** | 0.1494 | 1 | 0.6991 |
| **GLOBAL** | 0.3391 | 2 | 0.8441 |

# **3. Queen Survival - Colony Death Treated as Queen Failure**

The above models considered queen replacement event which did not
accompany the death of the colony. In other words, it considered only
successful queen replacement events. Many colonies which died became
queenless long before the last bees disappeared; such cases can be
thought of as failed queen replacement events. However we cannot
reliably distinguish all colonies that died from queen loss. The
following model is the same as the queen survival model except that all
colony deaths are counted as queen events.

**Model Code in R**

BHPtr<-coxph(Surv(tstart, tstop, Queen) ~ strata(Region) + Patties
+ Fume, Qsurv, cluster=Colony.Number)

**Analysis of Variance**

skipping term strata(Region)

Analysis of Deviance Table (Type II tests)


|  | Df | Chisq | Pr(>Chisq) |
| --- | --- | --- | --- |
| **Patties** | 1 | 6.355 | 0.0117 |
| **Fume** | 1 | 1.451 | 0.2284 |

**Effect Estimates**

Fitting Proportional Hazards Regression Model: Surv(tstart,
tstop, Queen) ~ strata(Region) + Patties + Fume


|  | coef | exp(coef) | robust se | z | p |
| --- | --- | --- | --- | --- | --- |
| **PattiesYes** | 0.231 | 1.26 | 0.09162 | 2.521 | 0.01 |
| **Fume** | -0.1385 | 0.8706 | 0.115 | -1.205 | 0.2 |

Likelihood ratio test=6.12 on 2 df, p=0.04690184 n= 2426, number of
events= 374

**Hazard Ratios**

| Patties effect | ratio | SE | df | asymp.LCL | asymp.UCL | null | z.ratio | p.value |
| --- | --- | --- | --- | --- | --- | --- | --- | --- |
| Yes / No | 1.26 | 0.1154 | Inf | 1.053 | 1.508 | 1 | 2.521 | 0.0117 |

| Fumagillin effect | ratio | SE | df | asymp.LCL | asymp.UCL | null | z.ratio | p.value |
| --- | --- | --- | --- | --- | --- | --- | --- | --- |
| Fume1 / Fume0 | 0.8706 | 0.1001 | Inf | 0.6949 | 1.091 | 1 | -1.205 | 0.2284 |

**Test of Hazard Proportionality**

|  | chisq | df | p |
| --- | --- | --- | --- |
| **Patties** | 0.4749 | 1 | 0.4907 |
| **Fume** | 0.2677 | 1 | 0.6049 |
| **GLOBAL** | 0.7552 | 2 | 0.6855 |

# **4. Adult Bee Population**

**Description**

This is a linear mixed effects model using the nlme package in R.
There are four fixed main effects (Date, Region Patties, and Fumagillin)
and one random effect (Colony). Successive observations on each colony
were expected to be correlated autoregressively. A different variance is
used for each region-date combination, because plots of residuals from
models without this adjustment were highly heteroskedastic. Data from
the first time point (“May 2014”) was excluded from the model because
there was no data for one region on that date.

Many variants of the model were considered, including

1. Splitting the “Date” term into two terms which accounted for length
   of treatment and seasonal effects separately. This would have allowed
   the May 2014 data to be included in the dataset.
2. Separate models within each region
3. Date, Region, and Apiary as random effects
4. Split colonies excluded, or the populations of the daughter colony
   attributed to the parent.
5. Different correlation terms, or no correlation term.

Results of these models informed comments in the text, but were not
dramatically different from those reported, and the alternatives were
inferior as judged by model fit criteria (Akaike Informative Criterion,
Bayesion Informative Criterion, likelihood ratio tests using maximum
likelihood prior to fixed effect reduction)

Because fumagillin was applied in fall 2014, that is, between the
third and fourth population assessement, effects of fumagillin are not
interpretable except in contrasts that specify the date term.

### **Full Model Code Prior to Fixed Effect Reduction**

bhpad<- lme(Adults ~ (Date+ Region+ Patties+ Fumagillin)^4 ,
random = ~1|Colony.Number, BHPad[BHPad$Date !=“May 2014”,],
correlation=corAR1(), weights=varIdent(form= ~1|Assessment\* Region),
method = “REML”, control=lmeControl(opt=“optim”))

### **Model Code as Reported**

bhpadr<-lme(Adults~(Date+ Region+ Patties+ Fumagillin) ^3 -
Date:Patties:Fumagillin - Date:Region:Patties, random =
~1|Colony.Number, BHPad[BHPad$Date !=“May 2014”,], correlation=corAR1(),
weights=varIdent(form= ~1|Assessment\* Region), method = “REML”,
control=lmeControl(opt=“optim”))

**Adult Bee Analysis of Variance**

|  | numDF | denDF | F-value | p-value |
| --- | --- | --- | --- | --- |
| **(Intercept)** | 1 | 1115 | 358.3 | 0 |
| **Date** | 5 | 1115 | 79.08 | 0 |
| **Region** | 2 | 350 | 29.9 | 1.024e-12 |
| **Patties** | 1 | 350 | 2.379 | 0.1239 |
| **Fumagillin** | 1 | 350 | 0.3897 | 0.5329 |
| **Date:Region** | 10 | 1115 | 33.45 | 0 |
| **Date:Patties** | 5 | 1115 | 2.996 | 0.01079 |
| **Date:Fumagillin** | 5 | 1115 | 0.952 | 0.4463 |
| **Region:Patties** | 2 | 350 | 0.3557 | 0.7009 |
| **Region:Fumagillin** | 2 | 350 | 0.9835 | 0.375 |
| **Patties:Fumagillin** | 1 | 350 | 0.0005378 | 0.9815 |
| **Date:Region:Fumagillin** | 10 | 1115 | 1.962 | 0.03411 |
| **Region:Patties:Fumagillin** | 2 | 350 | 3.408 | 0.03422 |

### **Contrasts and Estimated Marginal Means**

# 

#### **Protein Supplements Averaged Across All Regions and Dates After June 2014**

Only one comparison; significance threshold = 0.05.

The effect of Patties averaged over Fumagillin, Region, and
Dates after June 2014


| Contrast of Protein Treatments | estimate | SE | df | lower.CL | upper.CL | t.ratio | p.value |
| --- | --- | --- | --- | --- | --- | --- | --- |
| Yes - No | -1022 | 412.5 | 350 | -1833 | -210.6 | -2.477 | 0.01371 |

Estimated Marginal Means for Patties averaged over Fumagillin,
Region, and Dates after June 2014


| Protein Treatment | emmean | SE | df | lower.CL | upper.CL | t.ratio | p.value |
| --- | --- | --- | --- | --- | --- | --- | --- |
| No | 18307 | 291.2 | 350 | 17734 | 18879 | 62.87 | 8.907e-193 |
| Yes | 17285 | 314.1 | 350 | 16667 | 17902 | 55.03 | 2.158e-174 |

#### **Interaction of Protein Supplements and Date (Across Regions)**

All 6 comparisons are meaningful; significance threshold = 0.05/6 =
0.0083.

The effect of Patties averaged over Fumagillin and Region, by
Date


| Contrast of Protein Treatments | Date | estimate | SE | df | lower.CL | upper.CL | t.ratio | p.value |
| --- | --- | --- | --- | --- | --- | --- | --- | --- |
| Yes - No | June 2014 | 504.3 | 402.3 | 350 | -286.9 | 1296 | 1.254 | 0.2108 |
| Yes - No | August 2014 | -804.2 | 609.3 | 350 | -2003 | 394.2 | -1.32 | 0.1878 |
| Yes - No | May 2015 | -289.1 | 623.8 | 350 | -1516 | 937.8 | -0.4635 | 0.6433 |
| Yes - No | June 2015 | -1100 | 952.1 | 350 | -2973 | 772.4 | -1.156 | 0.2487 |
| Yes - No | August 2015 | -2150 | 649.7 | 350 | -3428 | -872.4 | -3.31 | 0.001032 |
| Yes - No | May 2016 | -765.7 | 792.8 | 350 | -2325 | 793.5 | -0.9659 | 0.3348 |

Estimated Marginal Means for Patty treatment groups averaged
over Fumagillin and Region, by Date


| Protein Treatment | Date | emmean | SE | df | lower.CL | upper.CL | t.ratio | p.value |
| --- | --- | --- | --- | --- | --- | --- | --- | --- |
| No | June 2014 | 9901 | 285.5 | 350 | 9340 | 10463 | 34.67 | 2.979e-115 |
| Yes | June 2014 | 10406 | 287.1 | 350 | 9841 | 10970 | 36.24 | 1.669e-120 |
| No | August 2014 | 17352 | 486.7 | 350 | 16395 | 18310 | 35.65 | 1.506e-118 |
| Yes | August 2014 | 16548 | 492.6 | 350 | 15579 | 17517 | 33.59 | 1.52e-111 |
| No | May 2015 | 13785 | 428.1 | 350 | 12943 | 14627 | 32.2 | 1.096e-106 |
| Yes | May 2015 | 13496 | 455.7 | 350 | 12599 | 14392 | 29.61 | 2.358e-97 |
| No | June 2015 | 21905 | 672.4 | 350 | 20583 | 23227 | 32.58 | 5.215e-108 |
| Yes | June 2015 | 20805 | 749.2 | 350 | 19331 | 22278 | 27.77 | 1.686e-90 |
| No | August 2015 | 26142 | 485.2 | 350 | 25188 | 27096 | 53.87 | 1.706e-171 |
| Yes | August 2015 | 23992 | 522.2 | 350 | 22965 | 25019 | 45.95 | 2.664e-150 |
| No | May 2016 | 12349 | 556.3 | 350 | 11255 | 13443 | 22.2 | 8.993e-69 |
| Yes | May 2016 | 11583 | 596.6 | 350 | 10410 | 12757 | 19.41 | 1.663e-57 |

#### **Interaction of Protein Supplements and Region (Across the 5 Dates after June 2014)**

All 3 comparison are meaningful; significance threshold = 0.05/3 =
0.0167.

The effect of Patties, by Region, last 5 dates of
Trial


| Contrast of Protein Treatments | Region | estimate | SE | df | lower.CL | upper.CL | t.ratio | p.value |
| --- | --- | --- | --- | --- | --- | --- | --- | --- |
| Yes - No | Southern Alberta | -337.4 | 525.5 | 350 | -1371 | 696.1 | -0.6421 | 0.5212 |
| Yes - No | Northern Alberta | -1763 | 682.3 | 350 | -3105 | -420.9 | -2.584 | 0.01018 |
| Yes - No | Prince Edward Island | -965.4 | 606.5 | 350 | -2158 | 227.5 | -1.592 | 0.1124 |

Estimated Marginal Means for Patties, by Region, Last 5 dates
of Trial


| Protein Treatments | Region | emmean | SE | df | lower.CL | upper.CL | t.ratio | p.value |
| --- | --- | --- | --- | --- | --- | --- | --- | --- |
| No | Southern Alberta | 22229 | 385.3 | 350 | 21471 | 22986 | 57.7 | 7.319e-181 |
| Yes | Southern Alberta | 21891 | 393.8 | 350 | 21117 | 22666 | 55.59 | 9.321e-176 |
| No | Northern Alberta | 21626 | 512.1 | 350 | 20619 | 22633 | 42.23 | 1.911e-139 |
| Yes | Northern Alberta | 19863 | 593.5 | 350 | 18696 | 21031 | 33.47 | 4.133e-111 |
| No | Prince Edward Island | 11065 | 517.8 | 350 | 10046 | 12083 | 21.37 | 1.994e-65 |
| Yes | Prince Edward Island | 10099 | 503.6 | 350 | 9109 | 11090 | 20.05 | 4.182e-60 |

#### **Effect of Patties Within Each Region and Date**

All 18 comparisons are meaningful; significance threshold = 0.05/18 =
0.00278.

The effect of Patties averaged over Fumagillin, by Region and
Date, from June 2014


| Contrast of Protein Treatments | Region | Date | estimate | SE | df | lower.CL | upper.CL | t.ratio | p.value |
| --- | --- | --- | --- | --- | --- | --- | --- | --- | --- |
| Yes - No | Southern Alberta | June 2014 | 1189 | 583.4 | 350 | 41.32 | 2336 | 2.038 | 0.04234 |
| Yes - No | Southern Alberta | August 2014 | -119.6 | 706.8 | 350 | -1510 | 1271 | -0.1693 | 0.8657 |
| Yes - No | Southern Alberta | May 2015 | 395.4 | 734.5 | 350 | -1049 | 1840 | 0.5383 | 0.5907 |
| Yes - No | Southern Alberta | June 2015 | -415.7 | 998.6 | 350 | -2380 | 1548 | -0.4163 | 0.6774 |
| Yes - No | Southern Alberta | August 2015 | -1466 | 691.1 | 350 | -2825 | -106.5 | -2.121 | 0.03463 |
| Yes - No | Southern Alberta | May 2016 | -81.22 | 856.4 | 350 | -1766 | 1603 | -0.09484 | 0.9245 |
| Yes - No | Northern Alberta | June 2014 | -236.7 | 668.9 | 350 | -1552 | 1079 | -0.3539 | 0.7236 |
| Yes - No | Northern Alberta | August 2014 | -1545 | 842.2 | 350 | -3202 | 111.3 | -1.835 | 0.06741 |
| Yes - No | Northern Alberta | May 2015 | -1030 | 805 | 350 | -2613 | 553.2 | -1.28 | 0.2015 |
| Yes - No | Northern Alberta | June 2015 | -1841 | 1091 | 350 | -3988 | 305.4 | -1.687 | 0.0925 |
| Yes - No | Northern Alberta | August 2015 | -2891 | 854 | 350 | -4571 | -1212 | -3.386 | 0.0007907 |
| Yes - No | Northern Alberta | May 2016 | -1507 | 957.4 | 350 | -3390 | 376.3 | -1.574 | 0.1165 |
| Yes - No | Prince Edward Island | June 2014 | 560.8 | 544.8 | 350 | -510.6 | 1632 | 1.03 | 0.3039 |
| Yes - No | Prince Edward Island | August 2014 | -747.6 | 709.9 | 350 | -2144 | 648.5 | -1.053 | 0.293 |
| Yes - No | Prince Edward Island | May 2015 | -232.6 | 760.8 | 350 | -1729 | 1264 | -0.3057 | 0.76 |
| Yes - No | Prince Edward Island | June 2015 | -1044 | 1063 | 350 | -3135 | 1047 | -0.9817 | 0.3269 |
| Yes - No | Prince Edward Island | August 2015 | -2094 | 811.5 | 350 | -3690 | -497.7 | -2.58 | 0.01028 |
| Yes - No | Prince Edward Island | May 2016 | -709.2 | 913.5 | 350 | -2506 | 1087 | -0.7764 | 0.4381 |

#### **Effect of Fumagillin Treatments, All Dates After the First Treatment, Combined**

Only one contrast; significance threshold =0.05.

The effect of Fumagillin averaged over Patties, Region, and
Date, for dates after the first treatment


| contrast | estimate | SE | df | lower.CL | upper.CL | t.ratio | p.value |
| --- | --- | --- | --- | --- | --- | --- | --- |
| Yes - No | 10.82 | 494.9 | 350 | -962.5 | 984.1 | 0.02186 | 0.9826 |

Estimated Marginal Means for Fumagillin averaged over Patties,
Region, and Date, for dates after the first treatment


| Fumagillin | emmean | SE | df | lower.CL | upper.CL | t.ratio | p.value |
| --- | --- | --- | --- | --- | --- | --- | --- |
| No | 18002 | 366.5 | 350 | 17281 | 18722 | 49.12 | 4.204e-159 |
| Yes | 18012 | 333.4 | 350 | 17357 | 18668 | 54.02 | 7.116e-172 |

#### **Effect of Fumagillin by Date**

Contrasts are meaningful beginning May 2015; significance threshold =
0.05/4= 0.0125.

The effect of Fumagillin averaged over Patties and Region, by
Date, after June 2014


| contrast | Date | estimate | SE | df | lower.CL | upper.CL | t.ratio | p.value |
| --- | --- | --- | --- | --- | --- | --- | --- | --- |
| Yes - No | August 2014 | -114.8 | 766.8 | 350 | -1623 | 1393 | -0.1497 | 0.8811 |
| Yes - No | May 2015 | -66.45 | 625.8 | 350 | -1297 | 1164 | -0.1062 | 0.9155 |
| Yes - No | June 2015 | -17.96 | 1053 | 350 | -2088 | 2052 | -0.01706 | 0.9864 |
| Yes - No | August 2015 | -1033 | 769.4 | 350 | -2546 | 479.9 | -1.343 | 0.1801 |
| Yes - No | May 2016 | 1161 | 836.6 | 350 | -484.5 | 2806 | 1.388 | 0.1661 |

Estimated Marginal Means for Fumagillin averaged over Patties
and Region, by Date, after June 2014


| Fumagillin | Date | emmean | SE | df | lower.CL | upper.CL | t.ratio | p.value |
| --- | --- | --- | --- | --- | --- | --- | --- | --- |
| No | August 2014 | 17008 | 552.4 | 350 | 15921 | 18094 | 30.79 | 1.234e-101 |
| Yes | August 2014 | 16893 | 531.7 | 350 | 15847 | 17939 | 31.77 | 3.728e-105 |
| No | May 2015 | 13673 | 459.8 | 350 | 12769 | 14578 | 29.74 | 7.999e-98 |
| Yes | May 2015 | 13607 | 425.2 | 350 | 12771 | 14443 | 32 | 5.792e-106 |
| No | June 2015 | 21364 | 784.8 | 350 | 19820 | 22907 | 27.22 | 2.019e-88 |
| Yes | June 2015 | 21346 | 705.8 | 350 | 19958 | 22734 | 30.24 | 1.158e-99 |
| No | August 2015 | 25583 | 563.7 | 350 | 24475 | 26692 | 45.39 | 1.066e-148 |
| Yes | August 2015 | 24550 | 524.7 | 350 | 23518 | 25582 | 46.79 | 1.105e-152 |
| No | May 2016 | 11386 | 634.1 | 350 | 10139 | 12633 | 17.96 | 1.442e-51 |
| Yes | May 2016 | 12547 | 546.9 | 350 | 11471 | 13622 | 22.94 | 9.667e-72 |

#### **Effect of Fumagillin by Region, Including Only Dates After the First Treatment**

All contrasts are meaningful; significance threshold = 0.05/3 =
0.0167.

The effect of Fumagillin averaged over Patties and Date, by
Region


| contrast | Region | estimate | SE | df | lower.CL | upper.CL | t.ratio | p.value |
| --- | --- | --- | --- | --- | --- | --- | --- | --- |
| Yes - No | Southern Alberta | 264.8 | 639.5 | 350 | -993 | 1523 | 0.4141 | 0.6791 |
| Yes - No | Northern Alberta | 892.8 | 923.3 | 350 | -923 | 2709 | 0.967 | 0.3342 |
| Yes - No | Prince Edward Island | -1125 | 971.4 | 350 | -3036 | 785.4 | -1.158 | 0.2475 |

Estimated Marginal Means for Fumagillin averaged over Patties
and Date, by Region


| Fumagillin | Region | emmean | SE | df | lower.CL | upper.CL | t.ratio | p.value |
| --- | --- | --- | --- | --- | --- | --- | --- | --- |
| No | Southern Alberta | 23428 | 468.3 | 350 | 22506 | 24349 | 50.02 | 1.626e-161 |
| Yes | Southern Alberta | 23692 | 435.4 | 350 | 22836 | 24549 | 54.42 | 7.407e-173 |
| No | Northern Alberta | 19236 | 684.8 | 350 | 17889 | 20583 | 28.09 | 1.057e-91 |
| Yes | Northern Alberta | 20129 | 623 | 350 | 18904 | 21355 | 32.31 | 4.622e-107 |
| No | Prince Edward Island | 11341 | 721.8 | 350 | 9921 | 12761 | 15.71 | 1.807e-42 |
| Yes | Prince Edward Island | 10216 | 649.9 | 350 | 8938 | 11494 | 15.72 | 1.668e-42 |

#### **Fumagillin by Region and Date**

All listed contrasts are meaningful; significance thresholed =
0.05/12 = .00417

The effect of Fumagillin averaged over Patties, by Region and
Date, after June 2014


| contrast | Region | Date | estimate | SE | df | lower.CL | upper.CL | t.ratio | p.value |
| --- | --- | --- | --- | --- | --- | --- | --- | --- | --- |
| Yes - No | Southern Alberta | May 2015 | 861.9 | 984.9 | 350 | -1075 | 2799 | 0.8751 | 0.3821 |
| Yes - No | Southern Alberta | June 2015 | 614.4 | 1250 | 350 | -1845 | 3074 | 0.4914 | 0.6234 |
| Yes - No | Southern Alberta | August 2015 | -1090 | 786.3 | 350 | -2637 | 456.5 | -1.386 | 0.1665 |
| Yes - No | Southern Alberta | May 2016 | 673 | 1104 | 350 | -1499 | 2845 | 0.6093 | 0.5427 |
| Yes - No | Northern Alberta | May 2015 | 689.2 | 1143 | 350 | -1558 | 2937 | 0.6032 | 0.5468 |
| Yes - No | Northern Alberta | June 2015 | 4423 | 1884 | 350 | 717.7 | 8128 | 2.348 | 0.01945 |
| Yes - No | Northern Alberta | August 2015 | -2263 | 1421 | 350 | -5058 | 532.7 | -1.592 | 0.1123 |
| Yes - No | Northern Alberta | May 2016 | 721.7 | 1587 | 350 | -2400 | 3843 | 0.4547 | 0.6496 |
| Yes - No | Prince Edward Island | May 2015 | -1750 | 1119 | 350 | -3952 | 450.8 | -1.564 | 0.1187 |
| Yes - No | Prince Edward Island | June 2015 | -5091 | 2205 | 350 | -9428 | -754.5 | -2.309 | 0.02153 |
| Yes - No | Prince Edward Island | August 2015 | 252.8 | 1640 | 350 | -2972 | 3477 | 0.1542 | 0.8776 |
| Yes - No | Prince Edward Island | May 2016 | 2088 | 1603 | 350 | -1064 | 5241 | 1.303 | 0.1935 |

#### **Fumagillin by Region, Date, and Patties**

These results are provided in support of figure 2 only. The four way
interaction was not significant in the analysis of variance. In keeping
with the presentation in that figure only contrasts between the
designated treatment group (here, fumagillin) and the totally untreated
control are considered. Also, measurement dates that precede the
treatment application are ignored. There are therefore (for Fumagillin),
12 meaningful contrasts and the threshold value is p=0.05/12 =
0.00417.

The effect of Fumagillin, by Date, Region, and
Patties


|  | Contrast of Fumagillin Treatment in Colonies Without Protein Supplements | Date | Region | Patties | estimate | SE | df | lower.CL | upper.CL | t.ratio | p.value |
| --- | --- | --- | --- | --- | --- | --- | --- | --- | --- | --- | --- |
| **3** | Yes - No | May 2015 | Southern Alberta | No | 850.7 | 1091 | 350 | -1294 | 2996 | 0.7801 | 0.4358 |
| **4** | Yes - No | June 2015 | Southern Alberta | No | 603.3 | 1333 | 350 | -2019 | 3225 | 0.4525 | 0.6512 |
| **5** | Yes - No | August 2015 | Southern Alberta | No | -1101 | 915.4 | 350 | -2902 | 699.2 | -1.203 | 0.2298 |
| **6** | Yes - No | May 2016 | Southern Alberta | No | 661.8 | 1204 | 350 | -1705 | 3029 | 0.5499 | 0.5828 |
| **15** | Yes - No | May 2015 | Northern Alberta | No | 2598 | 1245 | 350 | 148.8 | 5046 | 2.086 | 0.03768 |
| **16** | Yes - No | June 2015 | Northern Alberta | No | 6331 | 1938 | 350 | 2520 | 10143 | 3.267 | 0.001195 |
| **17** | Yes - No | August 2015 | Northern Alberta | No | -354.1 | 1491 | 350 | -3287 | 2578 | -0.2375 | 0.8124 |
| **18** | Yes - No | May 2016 | Northern Alberta | No | 2630 | 1654 | 350 | -622.3 | 5882 | 1.59 | 0.1126 |
| **27** | Yes - No | May 2015 | Prince Edward Island | No | -1617 | 1242 | 350 | -4059 | 824.5 | -1.303 | 0.1935 |
| **28** | Yes - No | June 2015 | Prince Edward Island | No | -4958 | 2267 | 350 | -9417 | -499.8 | -2.187 | 0.02939 |
| **29** | Yes - No | August 2015 | Prince Edward Island | No | 385.9 | 1724 | 350 | -3006 | 3777 | 0.2238 | 0.823 |
| **30** | Yes - No | May 2016 | Prince Edward Island | No | 2221 | 1687 | 350 | -1096 | 5539 | 1.317 | 0.1887 |

#### **Patties by Region, Date, and Fumagillin**

These results are provided in support of figure 2 only. The four way
interaction was not significant in the analysis of variance. In keeping
with the presentation in that figure only contrasts between the
designated treatment group (here, Patties) and the totally untreated
control are considered. Also, the first measurement date in each region
preceded the first treatment date. There are therefore (for Patties), 17
meaningful contrasts and the threshold value is p=0.05/17 = 0.00294.
However, only the 12 contrasts for dates after the first fumagillin
application are reflected on the graph. For earlier dates, see the
contrasts in the above table “Patties by Region and Date”.

The effect of Protein Supplements, by Date, Region, and
Patties


|  | Contrast of Protein Treatments in Colonies Without Fumagillin Treatments | Date | Region | Fumagillin | estimate | SE | df | lower.CL | upper.CL | t.ratio | p.value |
| --- | --- | --- | --- | --- | --- | --- | --- | --- | --- | --- | --- |
| **1** | Yes - No | June 2014 | Southern Alberta | No | 1178 | 763.6 | 350 | -324.1 | 2679 | 1.542 | 0.1239 |
| **2** | Yes - No | August 2014 | Southern Alberta | No | -130.8 | 861.4 | 350 | -1825 | 1563 | -0.1519 | 0.8794 |
| **3** | Yes - No | May 2015 | Southern Alberta | No | 384.2 | 888.7 | 350 | -1364 | 2132 | 0.4323 | 0.6658 |
| **4** | Yes - No | June 2015 | Southern Alberta | No | -426.9 | 1119 | 350 | -2627 | 1773 | -0.3816 | 0.703 |
| **5** | Yes - No | August 2015 | Southern Alberta | No | -1477 | 857.2 | 350 | -3163 | 209 | -1.723 | 0.08578 |
| **6** | Yes - No | May 2016 | Southern Alberta | No | -92.38 | 993.7 | 350 | -2047 | 1862 | -0.09297 | 0.926 |
| **13** | Yes - No | June 2014 | Northern Alberta | No | 1672 | 922.6 | 350 | -142.9 | 3486 | 1.812 | 0.07086 |
| **14** | Yes - No | August 2014 | Northern Alberta | No | 363.2 | 1058 | 350 | -1717 | 2444 | 0.3434 | 0.7315 |
| **15** | Yes - No | May 2015 | Northern Alberta | No | 878.3 | 1033 | 350 | -1153 | 2909 | 0.8505 | 0.3957 |
| **16** | Yes - No | June 2015 | Northern Alberta | No | 67.12 | 1270 | 350 | -2430 | 2564 | 0.05287 | 0.9579 |
| **17** | Yes - No | August 2015 | Northern Alberta | No | -982.9 | 1073 | 350 | -3093 | 1128 | -0.916 | 0.3603 |
| **18** | Yes - No | May 2016 | Northern Alberta | No | 401.6 | 1159 | 350 | -1878 | 2681 | 0.3465 | 0.7292 |
| **25** | Yes - No | June 2014 | Prince Edward Island | No | 694 | 768.2 | 350 | -816.9 | 2205 | 0.9034 | 0.3669 |
| **26** | Yes - No | August 2014 | Prince Edward Island | No | -614.5 | 898 | 350 | -2381 | 1152 | -0.6842 | 0.4943 |
| **27** | Yes - No | May 2015 | Prince Edward Island | No | -99.43 | 938.6 | 350 | -1945 | 1747 | -0.1059 | 0.9157 |
| **28** | Yes - No | June 2015 | Prince Edward Island | No | -910.6 | 1196 | 350 | -3263 | 1442 | -0.7612 | 0.447 |
| **29** | Yes - No | August 2015 | Prince Edward Island | No | -1961 | 976 | 350 | -3880 | -41.05 | -2.009 | 0.04532 |
| **30** | Yes - No | May 2016 | Prince Edward Island | No | -576 | 1068 | 350 | -2677 | 1525 | -0.5392 | 0.5901 |

#### **Patties + Fumagillin by Region & Date**

These results are provided in support of figure 2 only. The four way
interaction was not significant in the analysis of variance. In keeping
with the presentation in that figure only contrasts between the
designated treatment group (here, the double treatment of Patties and
Fumagillin) and the totally untreated control are considered. Also,
measurement dates that precede the fumagillin application are ignored.
There are therefore (for Patties + Fumagillin ), 12 meaningful contrasts
and the threshold value is p=0.05/12 = 0.00417.

The effect of the combined treatment of Protein and Fumagillin,
by Date, Region, and Patties


|  | Contrast of Both Treatments to No Treatment | Date | Region | estimate | SE | df | lower.CL | upper.CL | t.ratio | p.value |
| --- | --- | --- | --- | --- | --- | --- | --- | --- | --- | --- |
| **16** | Yes Yes - No No | May 2015 | Southern Alberta | 1257 | 1215 | 350 | -1133 | 3647 | 1.035 | 0.3015 |
| **22** | Yes Yes - No No | June 2015 | Southern Alberta | 198.7 | 1566 | 350 | -2882 | 3279 | 0.1269 | 0.8991 |
| **28** | Yes Yes - No No | August 2015 | Southern Alberta | -2556 | 1019 | 350 | -4560 | -551.3 | -2.508 | 0.01261 |
| **34** | Yes Yes - No No | May 2016 | Southern Alberta | 591.8 | 1407 | 350 | -2176 | 3360 | 0.4204 | 0.6744 |
| **52** | Yes Yes - No No | May 2015 | Northern Alberta | -340.9 | 1375 | 350 | -3045 | 2363 | -0.248 | 0.8043 |
| **58** | Yes Yes - No No | June 2015 | Northern Alberta | 2582 | 2155 | 350 | -1656 | 6820 | 1.198 | 0.2317 |
| **64** | Yes Yes - No No | August 2015 | Northern Alberta | -5154 | 1643 | 350 | -8385 | -1922 | -3.137 | 0.001853 |
| **70** | Yes Yes - No No | May 2016 | Northern Alberta | -785.1 | 1819 | 350 | -4362 | 2792 | -0.4317 | 0.6662 |
| **88** | Yes Yes - No No | May 2015 | Prince Edward Island | -1983 | 1385 | 350 | -4707 | 740.9 | -1.432 | 0.1531 |
| **94** | Yes Yes - No No | June 2015 | Prince Edward Island | -6135 | 2466 | 350 | -10986 | -1285 | -2.488 | 0.01332 |
| **100** | Yes Yes - No No | August 2015 | Prince Edward Island | -1841 | 1833 | 350 | -5446 | 1764 | -1.004 | 0.3159 |
| **106** | Yes Yes - No No | May 2016 | Prince Edward Island | 1379 | 1891 | 350 | -2340 | 5098 | 0.7293 | 0.4663 |

# **5. Sealed Brood Population**

The model for sealed brood was the same as that for adult bees,
except that the correlation term was dropped because it did not improve
the model fit statistics for brood.

This is a linear mixed effects model using the nlme package in R.
There are four fixed main effects (Date, Region Patties, and Fumagillin)
and one random effect (Colony). There were repeated observations on each
colony, which are expected to be correlated. A different variance is
used for each region-date combination. Data from the first time point
(“May 2014”) was excluded from the model because there was no data for
one region on that date.

Many variants of the model were considered, including

1. Splitting the “Date” term into two terms which accounted for length
   of treatment and seasonal effects seperately. This would have allowed
   the May 2014 data to be included in the dataset.
2. Seperate models within each region
3. Date, Region, and Apiary as random effects
4. Split colonies excluded, or the populations of the daughter colony
   attributed to the parent.

Results of these models informed comments in the text, but were not
dramatically different from those reported.

### **Full Model Code Before Fixed Effect Reduction**

bhpbr<-lme(Worker.Cells ~ (Region+ Date+ Patties+ Fumagillin)^4,
random = ~1|Colony.Number, BHPbr[BHPbr$Date !=“May 2014”,],
weights=varIdent(form= ~1|Assessment\* Region), method = “REML”)

### **Model Code as Reported**

bhpbr<-lme(Worker.Cells ~ (Region+ Date+ Patties+ Fumagillin)^3-
Date:Patties:Fumagillin- Region:Date:Patties, random = ~1|Colony.Number,
BHPbr[BHPbr$Date !=“May 2014”,], weights=varIdent(form= ~1|Assessment\*
Region), method = “REML”)

**Sealed Brood Analysis of Variance**

```
##                           numDF denDF  F-value p-value
## (Intercept)                   1  1091 324.0634  <.0001
## Region                        2   333   7.8414  0.0005
## Date                          5  1091  25.6827  <.0001
## Patties                       1   333  12.7855  0.0004
## Fumagillin                    1   333   0.2683  0.6048
## Region:Date                  10  1091  31.8632  <.0001
## Region:Patties                2   333   2.1491  0.1182
## Region:Fumagillin             2   333   0.6102  0.5439
## Date:Patties                  5  1091   5.3692  0.0001
## Date:Fumagillin               5  1091   0.4616  0.8050
## Patties:Fumagillin            1   333   0.0029  0.9573
## Region:Date:Fumagillin       10  1091   2.0106  0.0293
## Region:Patties:Fumagillin     2   333   4.1493  0.0166
```

### **Treatment Contrasts**

#### **Protein Supplements - Combined Dates After June 2014, Across Levels of Region and Fumagillin**

Only one contrast: significance threshold = 0.05

| Contrast of Protein Treatments | estimate | SE | df | lower.CL | upper.CL | t.ratio | p.value |
| --- | --- | --- | --- | --- | --- | --- | --- |
| Yes - No | -745.4 | 249.1 | 333 | -1235 | -255.5 | -2.993 | 0.00297 |

| Protein Treatment | emmean | SE | df | lower.CL | upper.CL | t.ratio | p.value |
| --- | --- | --- | --- | --- | --- | --- | --- |
| No | 9536 | 182.7 | 333 | 9176 | 9895 | 52.2 | 2.159e-162 |
| Yes | 8790 | 193.3 | 333 | 8410 | 9170 | 45.48 | 6.639e-145 |

#### **Protein Supplements After June 2014 - Combined Dates by Region**

There are three within region comparisons so the significance
threshold is 0.05/3 = 0.0167.

| Contrast of Protein Treatments | Region | estimate | SE | df | lower.CL | upper.CL | t.ratio | p.value |
| --- | --- | --- | --- | --- | --- | --- | --- | --- |
| Yes - No | Southern Alberta | -118.3 | 324.6 | 333 | -756.8 | 520.2 | -0.3645 | 0.7157 |
| Yes - No | Northern Alberta | -1245 | 411.8 | 333 | -2055 | -434.9 | -3.023 | 0.002697 |
| Yes - No | Prince Edward Island | -873 | 467.2 | 333 | -1792 | 46.08 | -1.868 | 0.06257 |

| Protein Treatment | Region | emmean | SE | df | lower.CL | upper.CL | t.ratio | p.value |
| --- | --- | --- | --- | --- | --- | --- | --- | --- |
| No | Southern Alberta | 10888 | 232.8 | 333 | 10430 | 11346 | 46.76 | 2.12e-148 |
| Yes | Southern Alberta | 10770 | 238.1 | 333 | 10301 | 11238 | 45.24 | 2.986e-144 |
| No | Northern Alberta | 9570 | 274.7 | 333 | 9030 | 10111 | 34.84 | 4.308e-113 |
| Yes | Northern Alberta | 8325 | 330 | 333 | 7676 | 8974 | 25.23 | 2.912e-79 |
| No | Prince Edward Island | 8149 | 397.4 | 333 | 7367 | 8931 | 20.5 | 5.31e-61 |
| Yes | Prince Edward Island | 7276 | 388.4 | 333 | 6512 | 8040 | 18.73 | 5.51e-54 |

#### **Interaction of Patties and Date - Across All Regions**

All six within date comparisons are meaningful, so the significance
threshold is 0.05/6 = 0.00833.

| Contrast of Protein Treatments | Date | estimate | SE | df | lower.CL | upper.CL | t.ratio | p.value |
| --- | --- | --- | --- | --- | --- | --- | --- | --- |
| Yes - No | June 2014 | 1328 | 397.3 | 333 | 546.2 | 2109 | 3.342 | 0.0009272 |
| Yes - No | August 2014 | -422.5 | 423.1 | 333 | -1255 | 409.7 | -0.9987 | 0.3187 |
| Yes - No | May 2015 | -168.4 | 376.8 | 333 | -909.6 | 572.7 | -0.447 | 0.6551 |
| Yes - No | June 2015 | -1293 | 507 | 333 | -2290 | -295.5 | -2.55 | 0.01122 |
| Yes - No | August 2015 | -649 | 396.9 | 333 | -1430 | 131.8 | -1.635 | 0.103 |
| Yes - No | May 2016 | -1194 | 579.8 | 333 | -2335 | -53.73 | -2.06 | 0.0402 |

| Protein Treatment | Date | emmean | SE | df | lower.CL | upper.CL | t.ratio | p.value |
| --- | --- | --- | --- | --- | --- | --- | --- | --- |
| No | June 2014 | 10288 | 290.8 | 333 | 9716 | 10860 | 35.38 | 7.74e-115 |
| Yes | June 2014 | 11616 | 292.8 | 333 | 11040 | 12192 | 39.67 | 3.137e-128 |
| No | August 2014 | 12129 | 368.5 | 333 | 11404 | 12854 | 32.91 | 1.042e-106 |
| Yes | August 2014 | 11706 | 376.8 | 333 | 10965 | 12447 | 31.07 | 2.055e-100 |
| No | May 2015 | 7659 | 259.5 | 333 | 7149 | 8170 | 29.52 | 5.556e-95 |
| Yes | May 2015 | 7491 | 277.9 | 333 | 6944 | 8038 | 26.96 | 1.025e-85 |
| No | June 2015 | 11731 | 368.5 | 333 | 11006 | 12456 | 31.83 | 4.916e-103 |
| Yes | June 2015 | 10438 | 406.2 | 333 | 9639 | 11237 | 25.7 | 4.88e-81 |
| No | August 2015 | 7807 | 282.9 | 333 | 7251 | 8364 | 27.59 | 4.818e-88 |
| Yes | August 2015 | 7158 | 299.4 | 333 | 6569 | 7747 | 23.91 | 2.917e-74 |
| No | May 2016 | 8351 | 462.7 | 333 | 7441 | 9261 | 18.05 | 2.857e-51 |
| Yes | May 2016 | 7157 | 489.2 | 333 | 6195 | 8119 | 14.63 | 8.792e-38 |

#### **Interaction of Patties and Date - By Region**

All 18 comparisons are meaningful, so the significance threshold is
0.05/18 = 0.00278.

| Contrast of Protein Treatments | Date | Region | estimate | SE | df | lower.CL | upper.CL | t.ratio | p.value |
| --- | --- | --- | --- | --- | --- | --- | --- | --- | --- |
| Yes - No | June 2014 | Southern Alberta | 1955 | 454.9 | 333 | 1060 | 2850 | 4.298 | 2.269e-05 |
| Yes - No | August 2014 | Southern Alberta | 204.6 | 433.1 | 333 | -647.4 | 1057 | 0.4725 | 0.6369 |
| Yes - No | May 2015 | Southern Alberta | 458.7 | 472 | 333 | -469.9 | 1387 | 0.9718 | 0.3319 |
| Yes - No | June 2015 | Southern Alberta | -665.7 | 540.9 | 333 | -1730 | 398.2 | -1.231 | 0.2192 |
| Yes - No | August 2015 | Southern Alberta | -21.87 | 448.1 | 333 | -903.2 | 859.5 | -0.04881 | 0.9611 |
| Yes - No | May 2016 | Southern Alberta | -567.2 | 620.2 | 333 | -1787 | 652.8 | -0.9145 | 0.3611 |
| Yes - No | June 2014 | Northern Alberta | 828.2 | 505.6 | 333 | -166.5 | 1823 | 1.638 | 0.1024 |
| Yes - No | August 2014 | Northern Alberta | -922.1 | 544.1 | 333 | -1992 | 148.2 | -1.695 | 0.09106 |
| Yes - No | May 2015 | Northern Alberta | -668 | 520.8 | 333 | -1692 | 356.4 | -1.283 | 0.2005 |
| Yes - No | June 2015 | Northern Alberta | -1792 | 601.4 | 333 | -2976 | -609.4 | -2.98 | 0.003091 |
| Yes - No | August 2015 | Northern Alberta | -1149 | 516.4 | 333 | -2164 | -132.7 | -2.224 | 0.02681 |
| Yes - No | May 2016 | Northern Alberta | -1694 | 643.5 | 333 | -2960 | -428.1 | -2.632 | 0.008874 |
| Yes - No | June 2014 | Prince Edward Island | 1200 | 564.1 | 333 | 90.57 | 2310 | 2.128 | 0.0341 |
| Yes - No | August 2014 | Prince Edward Island | -550.1 | 600.4 | 333 | -1731 | 631 | -0.9162 | 0.3602 |
| Yes - No | May 2015 | Prince Edward Island | -296 | 488.9 | 333 | -1258 | 665.7 | -0.6055 | 0.5453 |
| Yes - No | June 2015 | Prince Edward Island | -1420 | 651.3 | 333 | -2702 | -139.4 | -2.181 | 0.02987 |
| Yes - No | August 2015 | Prince Edward Island | -776.6 | 558.9 | 333 | -1876 | 322.8 | -1.39 | 0.1656 |
| Yes - No | May 2016 | Prince Edward Island | -1322 | 719.1 | 333 | -2736 | 92.59 | -1.838 | 0.0669 |

#### **Fumagillin After August 2014 - Regions Combined**

| Contrast of Fumagillin Treatments | estimate | SE | df | lower.CL | upper.CL | t.ratio | p.value |
| --- | --- | --- | --- | --- | --- | --- | --- |
| Yes - No | 104 | 304.5 | 333 | -495 | 702.9 | 0.3415 | 0.7329 |

| Fumagillin Treatment | emmean | SE | df | lower.CL | upper.CL | t.ratio | p.value |
| --- | --- | --- | --- | --- | --- | --- | --- |
| No | 8422 | 227.8 | 333 | 7974 | 8870 | 36.98 | 6.127e-120 |
| Yes | 8526 | 202.4 | 333 | 8128 | 8924 | 42.13 | 1.736e-135 |

#### **Fumagillin After August 2014 - by Region**

There are three within region comparisons so the significance
threshold is 0.05/3 = 0.0167.

| Contrast of Fumagillin Treatments | Region | estimate | SE | df | lower.CL | upper.CL | t.ratio | p.value |
| --- | --- | --- | --- | --- | --- | --- | --- | --- |
| Yes - No | Southern Alberta | 506.5 | 382 | 333 | -244.8 | 1258 | 1.326 | 0.1857 |
| Yes - No | Northern Alberta | 336.7 | 476.1 | 333 | -599.9 | 1273 | 0.7071 | 0.48 |
| Yes - No | Prince Edward Island | -531.3 | 679.8 | 333 | -1869 | 806 | -0.7815 | 0.4351 |

| Fumagillin Treatment | Region | emmean | SE | df | lower.CL | upper.CL | t.ratio | p.value |
| --- | --- | --- | --- | --- | --- | --- | --- | --- |
| No | Southern Alberta | 10659 | 278.3 | 333 | 10111 | 11206 | 38.3 | 4.649e-124 |
| Yes | Southern Alberta | 11165 | 261.6 | 333 | 10651 | 11680 | 42.67 | 4.741e-137 |
| No | Northern Alberta | 9216 | 353.2 | 333 | 8521 | 9911 | 26.1 | 1.62e-82 |
| Yes | Northern Alberta | 9553 | 320.7 | 333 | 8922 | 10183 | 29.79 | 6.119e-96 |
| No | Prince Edward Island | 5392 | 514.6 | 333 | 4380 | 6404 | 10.48 | 2.171e-22 |
| Yes | Prince Edward Island | 4861 | 444.1 | 333 | 3987 | 5734 | 10.95 | 5.062e-24 |

#### **Interaction of Fumagillin and Date - Across All Regions**

Four within date comparisons are meaningful, so the significance
threshold is 0.05/4 = 0.0125.

| Contrast of Fumagillin Treatments | Date | estimate | SE | df | lower.CL | upper.CL | t.ratio | p.value |
| --- | --- | --- | --- | --- | --- | --- | --- | --- |
| Yes - No | June 2014 | 302.1 | 427.5 | 333 | -538.8 | 1143 | 0.7066 | 0.4803 |
| Yes - No | August 2014 | 240.9 | 613.5 | 333 | -965.9 | 1448 | 0.3927 | 0.6948 |
| Yes - No | May 2015 | -14.67 | 382.7 | 333 | -767.5 | 738.2 | -0.03834 | 0.9694 |
| Yes - No | June 2015 | -623.6 | 584.5 | 333 | -1773 | 526.2 | -1.067 | 0.2868 |
| Yes - No | August 2015 | 0.6583 | 425.9 | 333 | -837.2 | 838.5 | 0.001546 | 0.9988 |
| Yes - No | May 2016 | 1054 | 754.2 | 333 | -430.1 | 2537 | 1.397 | 0.1634 |

| Fumagillin Treatment | Date | emmean | SE | df | lower.CL | upper.CL | t.ratio | p.value |
| --- | --- | --- | --- | --- | --- | --- | --- | --- |
| No | June 2014 | 10801 | 306.2 | 333 | 10199 | 11403 | 35.28 | 1.646e-114 |
| Yes | June 2014 | 11103 | 298.3 | 333 | 10516 | 11690 | 37.22 | 1.062e-120 |
| No | August 2014 | 11797 | 443.2 | 333 | 10925 | 12669 | 26.62 | 1.899e-84 |
| Yes | August 2014 | 12038 | 424.2 | 333 | 11203 | 12873 | 28.38 | 7.001e-91 |
| No | May 2015 | 7583 | 281.4 | 333 | 7029 | 8136 | 26.95 | 1.138e-85 |
| Yes | May 2015 | 7568 | 260 | 333 | 7056 | 8079 | 29.11 | 1.649e-93 |
| No | June 2015 | 11397 | 435.1 | 333 | 10541 | 12253 | 26.19 | 7.114e-83 |
| Yes | June 2015 | 10773 | 392.1 | 333 | 10002 | 11544 | 27.48 | 1.295e-87 |
| No | August 2015 | 7483 | 305.9 | 333 | 6881 | 8084 | 24.46 | 2.323e-76 |
| Yes | August 2015 | 7483 | 296.7 | 333 | 6899 | 8067 | 25.22 | 3.106e-79 |
| No | May 2016 | 7227 | 581.1 | 333 | 6084 | 8370 | 12.44 | 1.971e-29 |
| Yes | May 2016 | 8281 | 481.7 | 333 | 7333 | 9228 | 17.19 | 7.359e-48 |

**Fumagillin within Date and Region**

After the first fumagillin application there are four inspection
dates in each of the three regions (=12 comparisons within region and
date). Therefore the significance limit is 0.05/12 = 0.00417.

Effect of Fumagillin by Region and Date, not adjusted for
multiple comparisons, whole model


| Contrast of Fumagillin Treatments | Region | Date | estimate | SE | df | lower.CL | upper.CL | t.ratio | p.value |
| --- | --- | --- | --- | --- | --- | --- | --- | --- | --- |
| Yes - No | Southern Alberta | June 2014 | -340 | 547.2 | 333 | -1416 | 736.3 | -0.6214 | 0.5347 |
| Yes - No | Southern Alberta | August 2014 | 347 | 468.9 | 333 | -575.4 | 1269 | 0.7401 | 0.4598 |
| Yes - No | Southern Alberta | May 2015 | 588.1 | 627.6 | 333 | -646.4 | 1823 | 0.9371 | 0.3494 |
| Yes - No | Southern Alberta | June 2015 | 270.6 | 650.8 | 333 | -1010 | 1551 | 0.4158 | 0.6779 |
| Yes - No | Southern Alberta | August 2015 | 333.4 | 530.3 | 333 | -709.7 | 1377 | 0.6287 | 0.53 |
| Yes - No | Southern Alberta | May 2016 | 834.1 | 809.6 | 333 | -758.5 | 2427 | 1.03 | 0.3036 |
| Yes - No | Northern Alberta | June 2014 | -115.2 | 712.8 | 333 | -1517 | 1287 | -0.1617 | 0.8717 |
| Yes - No | Northern Alberta | August 2014 | -999.3 | 965 | 333 | -2898 | 899 | -1.036 | 0.3012 |
| Yes - No | Northern Alberta | May 2015 | 153.8 | 762.4 | 333 | -1346 | 1654 | 0.2017 | 0.8403 |
| Yes - No | Northern Alberta | June 2015 | 1415 | 943 | 333 | -439.5 | 3270 | 1.501 | 0.1343 |
| Yes - No | Northern Alberta | August 2015 | -920 | 746.6 | 333 | -2389 | 548.6 | -1.232 | 0.2187 |
| Yes - No | Northern Alberta | May 2016 | 697.5 | 883.5 | 333 | -1040 | 2435 | 0.7895 | 0.4304 |
| Yes - No | Prince Edward Island | June 2014 | 1361 | 915 | 333 | -438.4 | 3161 | 1.488 | 0.1377 |
| Yes - No | Prince Edward Island | August 2014 | 1375 | 1495 | 333 | -1567 | 4317 | 0.9194 | 0.3585 |
| Yes - No | Prince Edward Island | May 2015 | -785.9 | 586.4 | 333 | -1939 | 367.7 | -1.34 | 0.1811 |
| Yes - No | Prince Edward Island | June 2015 | -3557 | 1328 | 333 | -6168 | -945.4 | -2.679 | 0.007745 |
| Yes - No | Prince Edward Island | August 2015 | 588.6 | 890.8 | 333 | -1164 | 2341 | 0.6608 | 0.5092 |
| Yes - No | Prince Edward Island | May 2016 | 1629 | 1921 | 333 | -2151 | 5409 | 0.8478 | 0.3972 |

#### **Patties by Region, Date, and Fumagillin**

These results are provided in support of figure 3 only. The four way
interaction was not significant in the analysis of variance. In keeping
with the presentation in that figure only contrasts between the
designated treatment group (here, Patties) and the totally untreated
control are considered. Also, the first measurement date in each region
preceded the first treatment date. There are therefore (for Patties), 17
meaningful contrasts and the threshold value is p=0.05/17 = 0.00294.
However, only the 12 contrasts for dates after the first fumagillin
application are reflected on the graph. For earlier dates, see the
contrasts in the above table “Interaction of Patties and Date, by
Region”.

| Contrast of Protein Treatments in Colonies Without Fumagillin | Date | Region | Fumagillin | estimate | SE | df | lower.CL | upper.CL | t.ratio | p.value |
| --- | --- | --- | --- | --- | --- | --- | --- | --- | --- | --- |
| Yes - No | June 2014 | Southern Alberta | No | 1971 | 551.3 | 333 | 886.8 | 3056 | 3.576 | 0.000401 |
| Yes - No | August 2014 | Southern Alberta | No | 221 | 533.3 | 333 | -828.1 | 1270 | 0.4143 | 0.6789 |
| Yes - No | May 2015 | Southern Alberta | No | 475 | 569.5 | 333 | -645.2 | 1595 | 0.8342 | 0.4048 |
| Yes - No | June 2015 | Southern Alberta | No | -649.4 | 627.2 | 333 | -1883 | 584.3 | -1.035 | 0.3012 |
| Yes - No | August 2015 | Southern Alberta | No | -5.53 | 550 | 333 | -1088 | 1076 | -0.01005 | 0.992 |
| Yes - No | May 2016 | Southern Alberta | No | -550.9 | 696.7 | 333 | -1921 | 819.7 | -0.7907 | 0.4297 |
| Yes - No | June 2014 | Northern Alberta | No | 1679 | 652.3 | 333 | 395.7 | 2962 | 2.574 | 0.0105 |
| Yes - No | August 2014 | Northern Alberta | No | -71.44 | 686 | 333 | -1421 | 1278 | -0.1041 | 0.9171 |
| Yes - No | May 2015 | Northern Alberta | No | 182.6 | 669.3 | 333 | -1134 | 1499 | 0.2729 | 0.7851 |
| Yes - No | June 2015 | Northern Alberta | No | -941.8 | 734.2 | 333 | -2386 | 502.4 | -1.283 | 0.2005 |
| Yes - No | August 2015 | Northern Alberta | No | -297.9 | 667.8 | 333 | -1612 | 1016 | -0.4461 | 0.6558 |
| Yes - No | May 2016 | Northern Alberta | No | -843.3 | 774.2 | 333 | -2366 | 679.6 | -1.089 | 0.2768 |
| Yes - No | June 2014 | Prince Edward Island | No | 357.1 | 730.6 | 333 | -1080 | 1794 | 0.4888 | 0.6253 |
| Yes - No | August 2014 | Prince Edward Island | No | -1393 | 759.3 | 333 | -2887 | 100.5 | -1.835 | 0.06744 |
| Yes - No | May 2015 | Prince Edward Island | No | -1139 | 679.8 | 333 | -2476 | 198.1 | -1.676 | 0.09474 |
| Yes - No | June 2015 | Prince Edward Island | No | -2264 | 801.9 | 333 | -3841 | -686.1 | -2.823 | 0.005047 |
| Yes - No | August 2015 | Prince Edward Island | No | -1620 | 722.8 | 333 | -3041 | -197.9 | -2.241 | 0.02569 |
| Yes - No | May 2016 | Prince Edward Island | No | -2165 | 858.2 | 333 | -3853 | -476.9 | -2.523 | 0.01211 |

#### **Fumagillin by Region, Date, and Patties**

These results are provided in support of figure 3 only. The four way
interaction was not significant in the analysis of variance. In keeping
with the presentation in that figure only contrasts between the
designated treatment group (here, fumagillin) and the totally untreated
control are considered. Also, measurement dates that precede the
treatment application are ignored. There are therefore (for Fumagillin),
12 meaningful contrasts and the threshold value is p=0.05/12 =
0.00417.

|  | Contrast of Fumagillin Treatments in Colonies Without Protein Supplements | Date | Region | Patties | estimate | SE | df | lower.CL | upper.CL | t.ratio | p.value |
| --- | --- | --- | --- | --- | --- | --- | --- | --- | --- | --- | --- |
| **3** | Yes - No | May 2015 | Southern Alberta | No | 604.4 | 693.6 | 333 | -759.9 | 1969 | 0.8714 | 0.3841 |
| **4** | Yes - No | June 2015 | Southern Alberta | No | 286.9 | 714.5 | 333 | -1119 | 1692 | 0.4015 | 0.6883 |
| **5** | Yes - No | August 2015 | Southern Alberta | No | 349.7 | 608.8 | 333 | -847.7 | 1547 | 0.5745 | 0.566 |
| **6** | Yes - No | May 2016 | Southern Alberta | No | 850.4 | 863.3 | 333 | -847.8 | 2549 | 0.9851 | 0.3253 |
| **9** | Yes - No | May 2015 | Northern Alberta | No | 1004 | 822.3 | 333 | -613.2 | 2622 | 1.221 | 0.2228 |
| **10** | Yes - No | June 2015 | Northern Alberta | No | 2266 | 985.3 | 333 | 328 | 4204 | 2.3 | 0.02207 |
| **11** | Yes - No | August 2015 | Northern Alberta | No | -69.38 | 800.7 | 333 | -1644 | 1506 | -0.08665 | 0.931 |
| **12** | Yes - No | May 2016 | Northern Alberta | No | 1548 | 930.5 | 333 | -282.3 | 3379 | 1.664 | 0.0971 |
| **15** | Yes - No | May 2015 | Prince Edward Island | No | -1629 | 750 | 333 | -3104 | -153.7 | -2.172 | 0.03056 |
| **16** | Yes - No | June 2015 | Prince Edward Island | No | -4400 | 1397 | 333 | -7148 | -1651 | -3.149 | 0.001788 |
| **17** | Yes - No | August 2015 | Prince Edward Island | No | -254.5 | 1010 | 333 | -2241 | 1732 | -0.252 | 0.8012 |
| **18** | Yes - No | May 2016 | Prince Edward Island | No | 785.8 | 1972 | 333 | -3094 | 4666 | 0.3984 | 0.6906 |

#### **Patties + Fumagillin by Region & Date**

These results are provided in support of figure 3 only. The four way
interaction was not significant in the analysis of variance. In keeping
with the presentation in that figure only contrasts between the
designated treatment group (here, the double treatment of Patties and
Fumagillin) and the untreated control are considered. Also, measurement
dates that precede the fumagillin application are ignored. There are
therefore (for Patties + Fumagillin ), 12 meaningful contrasts and the
threshold value is p=0.05/12 = 0.00417.

|  | Contrast Between Colonies That Received Both Treatments and Colonies that Received Neither | Date | Region | estimate | SE | df | lower.CL | upper.CL | t.ratio | p.value |
| --- | --- | --- | --- | --- | --- | --- | --- | --- | --- | --- |
| **16** | Yes Yes - No No | May 2015 | Southern Alberta | 1047 | 775.8 | 333 | -479.3 | 2573 | 1.349 | 0.1782 |
| **22** | Yes Yes - No No | June 2015 | Southern Alberta | -395.2 | 834.2 | 333 | -2036 | 1246 | -0.4738 | 0.636 |
| **28** | Yes Yes - No No | August 2015 | Southern Alberta | 311.5 | 679.8 | 333 | -1026 | 1649 | 0.4583 | 0.6471 |
| **34** | Yes Yes - No No | May 2016 | Southern Alberta | 266.9 | 1014 | 333 | -1729 | 2262 | 0.2631 | 0.7927 |
| **52** | Yes Yes - No No | May 2015 | Northern Alberta | -514.2 | 907.8 | 333 | -2300 | 1272 | -0.5665 | 0.5715 |
| **58** | Yes Yes - No No | June 2015 | Northern Alberta | -377 | 1109 | 333 | -2559 | 1805 | -0.3398 | 0.7342 |
| **64** | Yes Yes - No No | August 2015 | Northern Alberta | -2069 | 896.7 | 333 | -3833 | -304.7 | -2.307 | 0.02167 |
| **70** | Yes Yes - No No | May 2016 | Northern Alberta | -996.4 | 1052 | 333 | -3065 | 1072 | -0.9475 | 0.3441 |
| **88** | Yes Yes - No No | May 2015 | Prince Edward Island | -1082 | 791.2 | 333 | -2638 | 474.6 | -1.367 | 0.1724 |
| **94** | Yes Yes - No No | June 2015 | Prince Edward Island | -4977 | 1490 | 333 | -7909 | -2046 | -3.34 | 0.0009339 |
| **100** | Yes Yes - No No | August 2015 | Prince Edward Island | -188 | 1055 | 333 | -2264 | 1888 | -0.1782 | 0.8587 |
| **106** | Yes Yes - No No | May 2016 | Prince Edward Island | 307 | 2076 | 333 | -3778 | 4392 | 0.1478 | 0.8826 |

# **6. Cluster Size**

### **Full Model**

seam<-lme(Seams~winter\* Month\* Region\* Patties+ winter\* Month\*
Region\* Fumagillin, data=clusterns , random = ~1 |Colony.Number,
correlation = corAR1(form = ~Assessment|Colony.Number),
na.action=na.omit, control=lmeControl(opt=“optim”),
weights=varIdent(form=~1|Region\* winter\* Month))

**Model Description**

Colonies which were split in August were excluded from the cluster
size dataset for the following winter because the recent split affected
the size and survival frequency. Colonies which were not viable in April
were also excluded from the dataset for the previous fall (that is, the
same colonies are being compared before and after winter, each year).
The interaction of patties and fumagillin was excluded from the model
because in PEI there were no non-split colonies in one treatment group
at the end of the study.

This is a linear mixed effects model using the nlme package in
R.There are five fixed effects: winter (1 or 2), Month (November or
April), Region (Southern Alberta, Northern Alberta, or Prince Edward
Island), Patties (Yes or No), and Fumagillin (Yes or No). The dependent
variable is cluster size, which is the estimated number of inter-frame
spaces filled with clustering bees during cold weather. Colony is a
random effect, and colonies are measured repeatedly; correlations are
expected between the measurements for a colony. Different variances are
permitted for each combination of region and date.

Alternative models were considered in depth and inform the text but
are not presented; these included

1. Different ways of accounting for split colonies (including them in
   the model, modelling them separately, or attributing all bees from the
   splits to the parent colony)
2. Separate analyses by region
3. Modelling the change in cluster size between November and April as
   the dependent variable, with or without initial cluster size as an
   additional independent variable.
4. Correlation structures other than AR1, nested random effects within
   apiary or region.

```
##                     numDF denDF   F-value p-value
## (Intercept)             1   552 1180.3087  <.0001
## winter                  1   552  193.7053  <.0001
## Month                   1   552   32.4561  <.0001
## Region                  2   235   30.0822  <.0001
## Patties                 1   235   13.3170  0.0003
## Fumagillin              1   235    0.3582  0.5501
## winter:Month            1   552   21.6662  <.0001
## winter:Region           2   552   28.8000  <.0001
## Month:Region            2   552   37.6568  <.0001
## winter:Patties          1   552    8.5863  0.0035
## Month:Patties           1   552    4.2353  0.0401
## Region:Patties          2   235    4.0028  0.0195
## winter:Month:Region     2   552   16.1278  <.0001
```

# Contrasts

The following table includes estimates for the effects of Patties
within region and date, even though treatment effects above two way
interactions were dropped from the model. Assuming this is acceptable,
all estimates are of interest; that is, 12 pairwise comparisons, so the
significance threshold is 0.05/12 = 0.004167.

Effect of Patties by Region and Date


| Contrast of Protein Treatments | Region | winter | Month | estimate | SE | df | t.ratio | p.value |
| --- | --- | --- | --- | --- | --- | --- | --- | --- |
| Yes - No | Southern Alberta | Winter 1 | November | 1.45 | 0.3974 | 235 | 3.649 | 0.0003241 |
| Yes - No | Southern Alberta | Winter 1 | April | 0.7788 | 0.4199 | 235 | 1.855 | 0.06486 |
| Yes - No | Southern Alberta | Winter 2 | November | 0.3451 | 0.3162 | 235 | 1.091 | 0.2762 |
| Yes - No | Southern Alberta | Winter 2 | April | -0.3262 | 0.4101 | 235 | -0.7955 | 0.4271 |
| Yes - No | Northern Alberta | Winter 1 | November | 0.358 | 0.4262 | 235 | 0.8399 | 0.4018 |
| Yes - No | Northern Alberta | Winter 1 | April | -0.3133 | 0.4191 | 235 | -0.7475 | 0.4555 |
| Yes - No | Northern Alberta | Winter 2 | November | -0.747 | 0.4669 | 235 | -1.6 | 0.111 |
| Yes - No | Northern Alberta | Winter 2 | April | -1.418 | 0.5116 | 235 | -2.772 | 0.006019 |
| Yes - No | Prince Edward Island | Winter 1 | November | -0.1106 | 0.6542 | 235 | -0.169 | 0.8659 |
| Yes - No | Prince Edward Island | Winter 1 | April | -0.7818 | 0.628 | 235 | -1.245 | 0.2144 |
| Yes - No | Prince Edward Island | Winter 2 | November | -1.216 | 0.6514 | 235 | -1.866 | 0.06327 |
| Yes - No | Prince Edward Island | Winter 2 | April | -1.887 | 0.6637 | 235 | -2.843 | 0.004866 |

Effect of Patties by Month


| Contrast of Protein Treatments | Month | estimate | SE | df | t.ratio | p.value |
| --- | --- | --- | --- | --- | --- | --- |
| Yes - No | November | 0.01334 | 0.2881 | 235 | 0.04629 | 0.9631 |
| Yes - No | April | -0.6579 | 0.3175 | 235 | -2.072 | 0.03933 |

Effect of Patties by winter


| Contrast of Protein Treatments | winter | estimate | SE | df | t.ratio | p.value |
| --- | --- | --- | --- | --- | --- | --- |
| Yes - No | Winter 1 | 0.2302 | 0.3043 | 235 | 0.7565 | 0.4501 |
| Yes - No | Winter 2 | -0.8748 | 0.3303 | 235 | -2.648 | 0.008634 |

Effect of Patties by Region


| Contrast of Protein Treatments | Region | estimate | SE | df | t.ratio | p.value |
| --- | --- | --- | --- | --- | --- | --- |
| Yes - No | Southern Alberta | 0.5619 | 0.2974 | 235 | 1.89 | 0.06003 |
| Yes - No | Northern Alberta | -0.5301 | 0.3836 | 235 | -1.382 | 0.1683 |
| Yes - No | Prince Edward Island | -0.9987 | 0.5997 | 235 | -1.665 | 0.09718 |

# **7. Change in Cluster Size During Winter**

**Maximum Likelihood Model**

seamd<-lme(seamdiff~(winter+ Region+ Patties)^3+ (winter+ Region+
Fumagillin)^3, data=clusternsdiff, random = ~1|Colony.Number,
na.action=na.omit, method= “ML”,
weights=varIdent(form=~1|Region\*winter))

**Model-as-Reported**

seamdr<-lme(seamdiff~winter\* Region+ Patties+ Fumagillin,
data=clusternsdiff, random = ~1|Colony.Number, na.action=na.omit,
method=“REML”, weights=varIdent(form=~1|Region\*winter))

**Analysis of Variance for Change in Cluster Size**

```
##               numDF denDF  F-value p-value
## (Intercept)       1   235 23.97310  <.0001
## winter            1   157 23.20977  <.0001
## Region            2   235 37.65695  <.0001
## Patties           1   235  3.72338  0.0549
## Fumagillin        1   235  0.75409  0.3861
## winter:Region     2   157 16.19584  <.0001
```

**Effect Estimates**

Effect of Patties


| Contrast of Protein Treatments | estimate | SE | df | t.ratio | p.value |
| --- | --- | --- | --- | --- | --- |
| Yes - No | 0.6569 | 0.3404 | 235 | 1.93 | 0.05486 |

# **8. Change in Cluster Size During Winter, Including Initial Cluster Size as a Fixed Effect**

**Full Model - Maximum Likelihood**

seamdc<-lme(seamdiff~(Seams+ winter+ Region+ Patties)^3+ (Seams+
winter+ Region+ Fumagillin)^3, data=clusternsdiff, random =
~1|Colony.Number, na.action=na.omit, method = “ML”,
weights=varIdent(form=~1|Region\*winter))

**Maximum Likelihood Comparison of Unreduced Model for Change
in Cluster Size, with and Without Initial Cluster Size in
Model**

```
##        Model df      AIC      BIC    logLik   Test  L.Ratio p-value
## seamd      1 25 2149.671 2249.457 -1049.835                        
## seamdc     2 39 2025.658 2181.325  -973.829 1 vs 2 152.0126  <.0001
```

The model that includes initial cluster size is clearly a better fit
to the data.

**Model-as-Reported**

```
##                     numDF denDF  F-value p-value
## (Intercept)             1   233 27.03861  <.0001
## Seams                   1   151 55.90187  <.0001
## winter                  1   151  0.47475  0.4919
## Region                  2   233  2.66059  0.0720
## Patties                 1   233  1.08661  0.2983
## Fumagillin              1   233  0.88133  0.3488
## Seams:winter            1   151  0.73838  0.3915
## Seams:Region            2   151  1.48345  0.2301
## winter:Region           2   151  0.91640  0.4022
## Region:Patties          2   233  3.09465  0.0472
## Seams:winter:Region     2   151  3.71118  0.0267
```

Main effect of protein supplements on change in cluster
size


| Contrast of Protein Treatments | estimate | SE | df | t.ratio | p.value |
| --- | --- | --- | --- | --- | --- |
| Yes - No | 0.337 | 0.3506 | 233 | 0.9611 | 0.3375 |

Interaction of protein supplements and region on change in
cluster size


| Contrast of Protein Treatments | Region | estimate | SE | df | t.ratio | p.value |
| --- | --- | --- | --- | --- | --- | --- |
| Yes - No | Northern Alberta | 1.125 | 0.4677 | 233 | 2.406 | 0.01692 |
| Yes - No | Southern Alberta | -0.4375 | 0.4197 | 233 | -1.042 | 0.2983 |
| Yes - No | Prince Edward Island | 0.3234 | 0.8445 | 233 | 0.3829 | 0.7021 |

The effect of initial cluster size, region, and year on the
change in cluster size during winter


| Contrast of hives with large (95th percentile) and small (5th percentile) initial cluster size | winter | Region | estimate | SE | df | t.ratio | p.value |
| --- | --- | --- | --- | --- | --- | --- | --- |
| Seams18.25 - Seams6.5125 | Winter 1 | Northern Alberta | 10.02 | 1.113 | 151 | 9.001 | 8.924e-16 |
| Seams18.25 - Seams6.5125 | Winter 2 | Northern Alberta | 8.003 | 2.05 | 151 | 3.905 | 0.0001419 |
| Seams18.25 - Seams6.5125 | Winter 1 | Southern Alberta | 7.826 | 1.047 | 151 | 7.477 | 5.769e-12 |
| Seams18.25 - Seams6.5125 | Winter 2 | Southern Alberta | 5.603 | 2.383 | 151 | 2.351 | 0.02001 |
| Seams18.25 - Seams6.5125 | Winter 1 | Prince Edward Island | 5.845 | 3.063 | 151 | 1.908 | 0.05825 |
| Seams18.25 - Seams6.5125 | Winter 2 | Prince Edward Island | 14.46 | 1.836 | 151 | 7.875 | 6.191e-13 |

# **9. Colony Weight**

### **Full Model**

cweight<-lme(Colony.Weight~winter\* Month\* Region\* Patties+ winter\*
Month\* Region\* Fumagillin, data=clusterns , random = ~1|Colony.Number,
na.action=na.omit, control=lmeControl(opt=“optim”),
weights=varIdent(form=~1|Region\* winter\* Month))

**Model Description**

Colonies which were split in August were excluded from the weight
dataset for the following winter because the recent split affected the
weight and survival frequency. Colonies which were not viable in April
were also excluded from the dataset for the previous fall (that is, the
same colonies are being compared before and after winter, each year).
The interaction of patties and fumagillin was excluded from the model
because in PEI there were no non-split colonies in one treatment group
at the end of the study.

This is a linear mixed effects model using the nlme package in
R.There are five fixed effects: winter (1 or 2), Month (November or
April), Region (Southern Alberta, Northern Alberta, or Prince Edward
Island), Patties (Yes or No), and Fumagillin (Yes or No). The dependent
variable is colony weight, which is the combined weight of bees, stored
feed, comb, and woodenware.Hive lids and bottom boards were not weighed
or their weight was subtracted from the total. Colony is a random
effect, and colonies are measured repeatedly; correlations are expected
between the measurements for a colony. Different variances are permitted
for each combination of region and date. The correlation term did not
contribute to the model fit and was dropped.

Alternative models were considered in depth and inform the text but
are not presented; these included

1. Different ways of accounting for split colonies (including them in
   the model or modelling them separately)
2. Separate analyses by region
3. Modelling the change in weight between November and April as the
   dependent variable, with or without initial cluster size as an
   additional independent variable.
4. Correlation structures other than AR1, nested random effects within
   apiary or region.

```
##                          numDF denDF  F-value p-value
## (Intercept)                  1   551 5463.417  <.0001
## winter                       1   551   11.677  0.0007
## Month                        1   551  488.096  <.0001
## Region                       2   235   59.915  <.0001
## Patties                      1   235   12.339  0.0005
## Fumagillin                   1   235   23.766  <.0001
## winter:Month                 1   551    8.353  0.0040
## winter:Region                2   551   65.120  <.0001
## Month:Region                 2   551    6.063  0.0025
## Month:Patties                1   551   15.693  0.0001
## winter:Fumagillin            1   551   27.576  <.0001
## Region:Fumagillin            2   235    5.735  0.0037
## winter:Month:Region          2   551    5.024  0.0069
## winter:Region:Fumagillin     2   551   10.710  <.0001
```

# Contrasts

Patties by Month


| Contrast of Protein Treatments | Month | estimate | SE | df | t.ratio | p.value |
| --- | --- | --- | --- | --- | --- | --- |
| Yes - No | November | 2.01 | 0.5721 | 235 | 3.513 | 0.0005318 |
| Yes - No | April | -0.08943 | 0.5405 | 235 | -0.1654 | 0.8687 |

Fumagillin by Winter and Region


| Contrast of Fumagillin Treatments | winter | Region | estimate | SE | df | t.ratio | p.value |
| --- | --- | --- | --- | --- | --- | --- | --- |
| Yes - No | Winter 1 | Southern Alberta | -4.208 | 0.8632 | 235 | -4.875 | 2.003e-06 |
| Yes - No | Winter 2 | Southern Alberta | -0.07936 | 0.7662 | 235 | -0.1036 | 0.9176 |
| Yes - No | Winter 1 | Northern Alberta | -0.1499 | 0.834 | 235 | -0.1797 | 0.8575 |
| Yes - No | Winter 2 | Northern Alberta | -1.565 | 1.032 | 235 | -1.516 | 0.1309 |
| Yes - No | Winter 1 | Prince Edward Island | -2.382 | 1.284 | 235 | -1.855 | 0.0648 |
| Yes - No | Winter 2 | Prince Edward Island | -5.327 | 3.446 | 235 | -1.546 | 0.1234 |

Fumagillin by Region


| Contrast of Fumagillin Treatments | Region | estimate | SE | df | t.ratio | p.value |
| --- | --- | --- | --- | --- | --- | --- |
| Yes - No | Southern Alberta | -2.144 | 0.7153 | 235 | -2.997 | 0.003017 |
| Yes - No | Northern Alberta | -0.8575 | 0.8023 | 235 | -1.069 | 0.2862 |
| Yes - No | Prince Edward Island | -3.855 | 1.879 | 235 | -2.052 | 0.04131 |

Winter, Month, and Region and their interactions are all highly
significant. Protein supplements had a significant interaction with
month.Supplemented colonies weighed 2 kg more than unsupplemented
colonies in November, but not in April. Fumagillin had a three way
interaction with winter and Region.Fumagillin treated colonies weighed
less on average in both years and in all regions, but the difference was
significant only in southern Alberta in the first winter. Fumagillin had
a significant interaction with Region. Fumagillin treated colonies
weighed less in all regions, but the difference was significant only in
Southern Alberta

The effect of removing splits and dead colonies from the dataset is
to clarify the effect of patties. It is an interaction with month, and
the regional differences are artefacts.

## 10. Change in Colony Weight During Winter

**Whole Model Without Split Colonies**

weightd<-lme(weightdiff~(winter+ Region+ Patties+ Fumagillin)^4,
data=clusternsdiff, random = ~1|Colony.Number, na.action=na.omit,
weights=varIdent(form=~1|Region\*winter))

anova(weightd, type = “marginal”)

```
##                          numDF denDF  F-value p-value
## (Intercept)                  1   230 666.9392  <.0001
## winter                       1   153   5.5542  0.0197
## Region                       2   230   1.4807  0.2296
## Patties                      1   230   8.6039  0.0037
## Fumagillin                   1   230   0.0007  0.9786
## winter:Region                2   153   4.5987  0.0115
## winter:Patties               1   153   0.5173  0.4731
## Region:Patties               2   230   0.1846  0.8315
## winter:Fumagillin            1   153   0.1364  0.7124
## Region:Fumagillin            2   230   0.7597  0.4690
## winter:Region:Patties        2   153   2.3927  0.0948
## winter:Region:Fumagillin     2   153   0.1321  0.8764
```

**Reduced Model**

```
##               numDF denDF   F-value p-value
## (Intercept)       1   234 1100.7069  <.0001
## winter            1   159   20.0577  <.0001
## Region            2   234    9.4545  0.0001
## Patties           1   234   21.2728  <.0001
## Fumagillin        1   234    0.0341  0.8536
## winter:Region     2   159    8.2226  0.0004
```

| Contrast of Protein Treatments | estimate | SE | df | t.ratio | p.value |
| --- | --- | --- | --- | --- | --- |
| Yes - No | 2.018 | 0.4375 | 234 | 4.612 | 6.56e-06 |

## **11. Change in Colony Weight, with Initial Cluster Size as a Factor in the Model**

**Full Model**

weighted<-lme(weightdiff~(Seams + winter+ Region+ Patties)^3+
(Seams + winter+ Region+ Fumagillin)^3, data=clusternsdiff, random =
~1|Colony.Number, na.action=na.omit,
weights=varIdent(form=~1|Region\*winter), method = “ML”)

**ANOVA**

**Reduced Model**

weighted<-lme(weightdiff~(winter+ Region+ Patties)^3+ Seams\*
Region+ Fumagillin, data=clusternsdiff, random = ~1|Colony.Number,
na.action=na.omit, weights=varIdent(form=~1|Region\*winter), method =
“REML”) anova(weighted, type = “marginal”)

```
##                       numDF denDF  F-value p-value
## (Intercept)               1   232 57.79801  <.0001
## winter                    1   152  0.08212  0.7748
## Region                    2   232  1.55049  0.2143
## Patties                   1   232  3.96377  0.0477
## Seams                     1   152 18.05499  <.0001
## Fumagillin                1   232  0.15723  0.6921
## winter:Region             2   152 15.47330  <.0001
## winter:Patties            1   152  2.01878  0.1574
## Region:Patties            2   232  0.33233  0.7176
## Region:Seams              2   152  4.42594  0.0135
## winter:Region:Patties     2   152  3.21035  0.0431
```

**Contrasts for Protein Treatments**

| Contrast of Protein Treatments | winter | Region | estimate | SE | df | t.ratio | p.value |
| --- | --- | --- | --- | --- | --- | --- | --- |
| Yes - No | Winter 1 | Southern Alberta | 1.278 | 0.6421 | 232 | 1.991 | 0.04766 |
| Yes - No | Winter 2 | Southern Alberta | 2.698 | 0.8846 | 152 | 3.05 | 0.002702 |
| Yes - No | Winter 1 | Northern Alberta | 2.009 | 0.8243 | 232 | 2.438 | 0.01554 |
| Yes - No | Winter 2 | Northern Alberta | -0.2181 | 1.164 | 152 | -0.1874 | 0.8516 |
| Yes - No | Winter 1 | Prince Edward Island | 2.347 | 1.825 | 232 | 1.286 | 0.1996 |
| Yes - No | Winter 2 | Prince Edward Island | -3.672 | 4.281 | 152 | -0.8577 | 0.3924 |

| Contrast of Protein Treatments | winter | estimate | SE | df | t.ratio | p.value |
| --- | --- | --- | --- | --- | --- | --- |
| Yes - No | Winter 1 | 1.878 | 0.7009 | 232 | 2.68 | 0.007894 |
| Yes - No | Winter 2 | -0.3973 | 1.508 | 152 | -0.2636 | 0.7925 |

| Contrast of Protein Treatments | Region | estimate | SE | df | t.ratio | p.value |
| --- | --- | --- | --- | --- | --- | --- |
| Yes - No | Southern Alberta | 1.988 | 0.5898 | 152 | 3.371 | 0.0009493 |
| Yes - No | Northern Alberta | 0.8955 | 0.7508 | 152 | 1.193 | 0.2348 |
| Yes - No | Prince Edward Island | -0.6623 | 2.323 | 152 | -0.2851 | 0.776 |

| Contrast of Protein Treatments | estimate | SE | df | t.ratio | p.value |
| --- | --- | --- | --- | --- | --- |
| Yes - No | 0.7405 | 0.837 | 152 | 0.8847 | 0.3777 |

**Effect of Colony Size, by Region**

# **12. Honey Production**

### **Full Model**

fm\_hp4 <- lme(Honey ~ (Region + Date) \* Patties \* Fumagillin +
Region \* Date, Xhp, random = ~ 1 | Colony.Number, weights =
varIdent(form = ~ 1 | Region \* Date))

**Model Description**

Honey production was measured only in Northern and Southern Alberta.
This is a linear mixed effects model using the nlme package in R and the
dependent variable (Honey) is the total surplus honey crop produced per
year. There are 4 fixed effects (Region: Southern Alberta and Northern
Alberta, Date:2014 and 2015, Patties: Yes and No, Fumagillin: Yes and
No). Colony is a random effect. Different variances are permitted for
each region:date combination. Since fumagillin was not applied until
fall 2014, results for 2014 reflect the effects of region and patties
only.

```
##                           numDF denDF   F-value p-value
## (Intercept)                   1   228  75.27394  <.0001
## Region                        1   228 145.08450  <.0001
## Date                          1   177  16.25318  0.0001
## Patties                       1   228   0.15789  0.6915
## Fumagillin                    1   228   0.00000  0.9994
## Region:Patties                1   228   1.49808  0.2222
## Date:Patties                  1   177   1.26033  0.2631
## Region:Fumagillin             1   228   6.01811  0.0149
## Date:Fumagillin               1   177   0.17967  0.6722
## Patties:Fumagillin            1   228   0.18267  0.6695
## Region:Date                   1   177 119.35054  <.0001
## Region:Patties:Fumagillin     1   228   6.29014  0.0128
## Date:Patties:Fumagillin       1   177   0.37069  0.5434
```

The effect of Region (contrast) on honey production in 2014 and
2015


| contrast | Date | estimate | SE | df | lower.CL | upper.CL | t.ratio | p.value |
| --- | --- | --- | --- | --- | --- | --- | --- | --- |
| Northern Alberta - Southern Alberta | August 2014 | 85.68 | 3.625 | 228 | 78.53 | 92.82 | 23.64 | 3.04e-63 |
| Northern Alberta - Southern Alberta | August 2015 | 20.42 | 5.176 | 177 | 10.2 | 30.63 | 3.945 | 0.000115 |

The effect of protein supplements on honey production in 2014
and 2015


| Contrast of Protein Treatments | Date | estimate | SE | df | lower.CL | upper.CL | t.ratio | p.value |
| --- | --- | --- | --- | --- | --- | --- | --- | --- |
| Yes - No | August 2014 | -1.952 | 3.289 | 228 | -8.432 | 4.529 | -0.5934 | 0.5535 |
| Yes - No | August 2015 | -6.261 | 4.461 | 177 | -15.06 | 2.542 | -1.404 | 0.1622 |

The effect of Fumagillin (contrast) in 2015 by Region averaged
over Patties


| Contrast of Fumagillin Treatments | Region | Date | estimate | SE | df | lower.CL | upper.CL | t.ratio | p.value |
| --- | --- | --- | --- | --- | --- | --- | --- | --- | --- |
| Yes - No | Southern Alberta | August 2015 | 3.826 | 4.435 | 177 | -4.927 | 12.58 | 0.8626 | 0.3895 |
| Yes - No | Northern Alberta | August 2015 | 9.245 | 6.257 | 177 | -3.103 | 21.59 | 1.478 | 0.1413 |

The effect of Fumagillin (contrast) in 2015 by Region and
Patties


| Contrast of Fumagillin Treatments | Region | Date | Patties | estimate | SE | df | lower.CL | upper.CL | t.ratio | p.value |
| --- | --- | --- | --- | --- | --- | --- | --- | --- | --- | --- |
| Yes - No | Southern Alberta | August 2015 | No | 2.459 | 6.131 | 177 | -9.641 | 14.56 | 0.401 | 0.6889 |
| Yes - No | Southern Alberta | August 2015 | Yes | 5.193 | 6.411 | 177 | -7.459 | 17.85 | 0.81 | 0.419 |
| Yes - No | Northern Alberta | August 2015 | No | 23.53 | 8.355 | 177 | 7.043 | 40.02 | 2.816 | 0.005407 |
| Yes - No | Northern Alberta | August 2015 | Yes | -5.041 | 9.315 | 177 | -23.42 | 13.34 | -0.5412 | 0.5891 |

The effect of Patties (contrast) by Region and averaged over
Fumagillin in 2014 and in 2015


| Contrast of Protein Treatments | Region | Date | estimate | SE | df | lower.CL | upper.CL | t.ratio | p.value |
| --- | --- | --- | --- | --- | --- | --- | --- | --- | --- |
| Yes - No | Southern Alberta | August 2014 | 0.4143 | 2.818 | 228 | -5.138 | 5.966 | 0.147 | 0.8832 |
| Yes - No | Southern Alberta | August 2015 | -3.895 | 4.439 | 177 | -12.66 | 4.865 | -0.8774 | 0.3814 |
| Yes - No | Northern Alberta | August 2014 | -4.317 | 5.775 | 228 | -15.7 | 7.063 | -0.7475 | 0.4555 |
| Yes - No | Northern Alberta | August 2015 | -8.627 | 6.305 | 177 | -21.07 | 3.816 | -1.368 | 0.173 |

The effect of Patties (contrast) in 2015 by Region and
Fumagillin


| contrast | Region | Date | Fumagillin | estimate | SE | df | lower.CL | upper.CL | t.ratio | p.value |
| --- | --- | --- | --- | --- | --- | --- | --- | --- | --- | --- |
| Yes - No | Southern Alberta | August 2015 | No | -5.262 | 6.434 | 177 | -17.96 | 7.435 | -0.8179 | 0.4145 |
| Yes - No | Southern Alberta | August 2015 | Yes | -2.528 | 6.113 | 177 | -14.59 | 9.536 | -0.4135 | 0.6797 |
| Yes - No | Northern Alberta | August 2015 | No | 5.659 | 9.021 | 177 | -12.14 | 23.46 | 0.6274 | 0.5312 |
| Yes - No | Northern Alberta | August 2015 | Yes | -22.91 | 8.74 | 177 | -40.16 | -5.664 | -2.621 | 0.009517 |

**Contrasts of each treatment group to all others within
Northern Alberta in 2015**

These are shown only in support of Fig 9, where the contrasts of
interest are the within-region contrasts in 2015 between the group that
received neither treatment and each of the other three groups.In the
context of the figure, there are six such contrasts, three for each
region, so the Bonferroni adjusted significance threshold is
0.05/6=0.0083.

```
##                           numDF denDF   F-value p-value
## (Intercept)                   1   228  75.27394  <.0001
## Region                        1   228 145.08450  <.0001
## Date                          1   177  16.25318  0.0001
## Patties                       1   228   0.15789  0.6915
## Fumagillin                    1   228   0.00000  0.9994
## Region:Patties                1   228   1.49808  0.2222
## Date:Patties                  1   177   1.26033  0.2631
## Region:Fumagillin             1   228   6.01811  0.0149
## Date:Fumagillin               1   177   0.17967  0.6722
## Patties:Fumagillin            1   228   0.18267  0.6695
## Region:Date                   1   177 119.35054  <.0001
## Region:Patties:Fumagillin     1   228   6.29014  0.0128
## Date:Patties:Fumagillin       1   177   0.37069  0.5434
```

The effect of Region (contrast) on honey production in 2014 and
2015


| contrast | Date | estimate | SE | df | lower.CL | upper.CL | t.ratio | p.value |
| --- | --- | --- | --- | --- | --- | --- | --- | --- |
| Northern Alberta - Southern Alberta | August 2014 | 85.68 | 3.625 | 228 | 78.53 | 92.82 | 23.64 | 3.04e-63 |
| Northern Alberta - Southern Alberta | August 2015 | 20.42 | 5.176 | 177 | 10.2 | 30.63 | 3.945 | 0.000115 |

The effect of protein supplements on honey production in 2014
and 2015


| Contrast of Protein Treatments | Date | estimate | SE | df | lower.CL | upper.CL | t.ratio | p.value |
| --- | --- | --- | --- | --- | --- | --- | --- | --- |
| Yes - No | August 2014 | -1.952 | 3.289 | 228 | -8.432 | 4.529 | -0.5934 | 0.5535 |
| Yes - No | August 2015 | -6.261 | 4.461 | 177 | -15.06 | 2.542 | -1.404 | 0.1622 |

The effect of Fumagillin (contrast) in 2015 by Region averaged
over Patties


| Contrast of Fumagillin Treatments | Region | Date | estimate | SE | df | lower.CL | upper.CL | t.ratio | p.value |
| --- | --- | --- | --- | --- | --- | --- | --- | --- | --- |
| Yes - No | Southern Alberta | August 2015 | 3.826 | 4.435 | 177 | -4.927 | 12.58 | 0.8626 | 0.3895 |
| Yes - No | Northern Alberta | August 2015 | 9.245 | 6.257 | 177 | -3.103 | 21.59 | 1.478 | 0.1413 |

The effect of Fumagillin (contrast) in 2015 by Region and
Patties


| Contrast of Fumagillin Treatments | Region | Date | Patties | estimate | SE | df | lower.CL | upper.CL | t.ratio | p.value |
| --- | --- | --- | --- | --- | --- | --- | --- | --- | --- | --- |
| Yes - No | Southern Alberta | August 2015 | No | 2.459 | 6.131 | 177 | -9.641 | 14.56 | 0.401 | 0.6889 |
| Yes - No | Southern Alberta | August 2015 | Yes | 5.193 | 6.411 | 177 | -7.459 | 17.85 | 0.81 | 0.419 |
| Yes - No | Northern Alberta | August 2015 | No | 23.53 | 8.355 | 177 | 7.043 | 40.02 | 2.816 | 0.005407 |
| Yes - No | Northern Alberta | August 2015 | Yes | -5.041 | 9.315 | 177 | -23.42 | 13.34 | -0.5412 | 0.5891 |

The effect of Patties (contrast) by Region and averaged over
Fumagillin in 2014 and in 2015


| Contrast of Protein Treatments | Region | Date | estimate | SE | df | lower.CL | upper.CL | t.ratio | p.value |
| --- | --- | --- | --- | --- | --- | --- | --- | --- | --- |
| Yes - No | Southern Alberta | August 2014 | 0.4143 | 2.818 | 228 | -5.138 | 5.966 | 0.147 | 0.8832 |
| Yes - No | Southern Alberta | August 2015 | -3.895 | 4.439 | 177 | -12.66 | 4.865 | -0.8774 | 0.3814 |
| Yes - No | Northern Alberta | August 2014 | -4.317 | 5.775 | 228 | -15.7 | 7.063 | -0.7475 | 0.4555 |
| Yes - No | Northern Alberta | August 2015 | -8.627 | 6.305 | 177 | -21.07 | 3.816 | -1.368 | 0.173 |

The effect of Patties (contrast) in 2015 by Region and
Fumagillin


| contrast | Region | Date | Fumagillin | estimate | SE | df | lower.CL | upper.CL | t.ratio | p.value |
| --- | --- | --- | --- | --- | --- | --- | --- | --- | --- | --- |
| Yes - No | Southern Alberta | August 2015 | No | -5.262 | 6.434 | 177 | -17.96 | 7.435 | -0.8179 | 0.4145 |
| Yes - No | Southern Alberta | August 2015 | Yes | -2.528 | 6.113 | 177 | -14.59 | 9.536 | -0.4135 | 0.6797 |
| Yes - No | Northern Alberta | August 2015 | No | 5.659 | 9.021 | 177 | -12.14 | 23.46 | 0.6274 | 0.5312 |
| Yes - No | Northern Alberta | August 2015 | Yes | -22.91 | 8.74 | 177 | -40.16 | -5.664 | -2.621 | 0.009517 |

**Contrasts of each treatment group to all others within
Northern Alberta in 2015**

These are shown only in support of Fig 9, where the contrasts of
interest are the within-region contrasts in 2015 between the group that
received neither treatment and each of the other three groups.In the
context of the figure, there are six such contrasts, three for each
region, so the Bonferroni adjusted significance threshold is
0.05/6=0.0083.

The contrasts among treatment groups in 2015


|  | Contrast of Treatment Groups (Patties:Fumagillin) Against the Untreated Group | Date | Region | estimate | SE | df | lower.CL | upper.CL | t.ratio | p.value |
| --- | --- | --- | --- | --- | --- | --- | --- | --- | --- | --- |
| **1** | Yes No - No No | August 2015 | Southern Alberta | -5.262 | 6.434 | 177 | -17.96 | 7.435 | -0.8179 | 0.4145 |
| **2** | No Yes - No No | August 2015 | Southern Alberta | 2.459 | 6.131 | 177 | -9.641 | 14.56 | 0.401 | 0.6889 |
| **4** | Yes Yes - No No | August 2015 | Southern Alberta | -0.06907 | 6.13 | 177 | -12.17 | 12.03 | -0.01127 | 0.991 |
| **7** | Yes No - No No | August 2015 | Northern Alberta | 5.659 | 9.021 | 177 | -12.14 | 23.46 | 0.6274 | 0.5312 |
| **8** | No Yes - No No | August 2015 | Northern Alberta | 23.53 | 8.355 | 177 | 7.043 | 40.02 | 2.816 | 0.005407 |
| **10** | Yes Yes - No No | August 2015 | Northern Alberta | 0.6185 | 8.805 | 177 | -16.76 | 18 | 0.07024 | 0.9441 |

# **13. Pollen Collection 2014**

### **Full Model**

pol14ndr<-lme(sqrt(Total.Pollen.per.Day) ~ Date + Patty\* Region,
random = ~1|Apiary, data=p14, weights = varIdent( form = ~1|Date\*
Region), method = “REML”)

**Model Description**

This is a linear mixed effects model using the nlme package from R.
In 2014, pollen was collected once from each colony in Northern and
Southern Alberta. The dependent variable in the model is the square root
of the amount of pollen per day collected by the colony. The square root
transformation was used because the residuals from untransformed models
had a skewed distribution and deviated from the normal quantile
function. Since fumagillin had not yet been applied in 2014, there are
only three fixed effects: Date (Various between July 1 and August 8,
2014), Region (Southern or Northern Alberta), and Patty (Yes or No).
Since each colony was measured only once, apiary is used as the random
term. Different variances are used for each region and date.

The pollen collections were conducted during the canola bloom. The
experimental design was that protein supplements were applied throughout
the active season except during the canola bloom, so this model tests
the effect of recent (but not current) protein supplementation on pollen
foraging activity.

```
##                   numDF denDF   F-value p-value
## (Intercept)           1   223 105.94351  <.0001
## Pollen.Dates.2014     6   223  29.80784  <.0001
## Patties               1   223   4.78952  0.0297
## Region                1     4  10.66835  0.0309
## Patties:Region        1   223   5.98105  0.0152
```

Back-transformed approximate treatment effect of Patties by
Region averaged over the dates


| Contrast of Protein Treatments | Region | estimate | SE | df | lower.CL | upper.CL | t.ratio | p.value |
| --- | --- | --- | --- | --- | --- | --- | --- | --- |
| Yes - No | Southern Alberta | -2.385 | 1.062 | 5 | -5.114 | 0.3449 | -2.246 | 0.07467 |
| Yes - No | Northern Alberta | 2.12 | 1.665 | 4 | -2.502 | 6.743 | 1.273 | 0.2718 |

Pollen Collection Emmeans by Region


| Patties | Region | response | SE | df | lower.CL | upper.CL | t.ratio | p.value |
| --- | --- | --- | --- | --- | --- | --- | --- | --- |
| No | Southern Alberta | 9.252 | 1.01 | 5 | 6.656 | 11.85 | 9.16 | 0.0002599 |
| Yes | Southern Alberta | 6.868 | 0.8379 | 5 | 4.714 | 9.022 | 8.196 | 0.00044 |
| No | Northern Alberta | 15.74 | 1.509 | 4 | 11.55 | 19.93 | 10.43 | 0.0004768 |
| Yes | Northern Alberta | 17.86 | 1.569 | 4 | 13.51 | 22.22 | 11.38 | 0.0003396 |

Back-transformed approximate treatment effect of Patties by
Region averaged over the dates


| Contrast of Regions | estimate | SE | df | lower.CL | upper.CL | t.ratio | p.value |
| --- | --- | --- | --- | --- | --- | --- | --- |
| Northern Alberta - Southern Alberta | 8.742 | 1.663 | 4 | 4.127 | 13.36 | 5.259 | 0.006261 |

Back-transformed approximate treatment effect of Patties by
Region averaged over the dates


| contrast | estimate | SE | df | lower.CL | upper.CL | t.ratio | p.value |
| --- | --- | --- | --- | --- | --- | --- | --- |
| Yes - No | -0.1323 | 0.9879 | 4 | -2.875 | 2.61 | -0.1339 | 0.8999 |

# **14. Pollen Collection 2015**

### **Full Model**

mmsr1 <- lme(sqrt(Pollen.per.Day) ~ (Region + Season + Patty) ^ 2,
random = ~ 1 | Colony, data = p15m, method = “REML”, weights =
varIdent(form = ~ 1 | Apiary \* Season),  
control = lmeControl(opt = “optim”))

**Model Description**

This is a linear mixed effects model using the nlme package from R.
In 2015, pollen was collected approximately every two weeks from the
same subset of colonies in each apiary. The dependent variable in the
model is the square root of the amount of pollen per day collected by
the colony. The square root transformation was used because the
residuals from untransformed models had a skewed distribution and
deviated from the normal quantile function. Prior fumagillin treatment
was not expected to influence pollen collection, and did not, so it was
dropped from the model. Because colonies were sampled repeatedly, colony
was used as the random term in the model. The quantity of pollen
collected is non-linearly related to date and varies greatly from day to
day. Therefore the date term was simplified by collapsing it into three
categories: early, mid, and late season. For Alberta colonies, the mid
season corresponds to the canola bloom, when protein supplements were
not applied.

```
##               numDF denDF  F-value p-value
## (Intercept)       1   560 420.5204  <.0001
## Region            2    91  15.5365  <.0001
## Season            2   560  22.1121  <.0001
## Patty             1    91   0.8012  0.3731
## Region:Season     4   560  52.3025  <.0001
## Region:Patty      2    91   0.5565  0.5752
## Season:Patty      2   560   3.3697  0.0351
```

Back-transformed approximate treatment effect of Patty by
Season and averaged over Region


| contrast | Season | estimate | SE | df | lower.CL | upper.CL | t.ratio | p.value |
| --- | --- | --- | --- | --- | --- | --- | --- | --- |
| yes patty - no patty | Early | -3.034 | 2.166 | 91 | -7.335 | 1.268 | -1.401 | 0.1646 |
| yes patty - no patty | Late | 2.581 | 1.454 | 91 | -0.3073 | 5.47 | 1.775 | 0.07923 |
| yes patty - no patty | Mid | -2.441 | 1.873 | 91 | -6.162 | 1.28 | -1.303 | 0.1958 |

Back-transformed approximate treatment effect of Region by
Season and averaged over Patty


| contrast | Season | estimate | SE | df | lower.CL | upper.CL | t.ratio | p.value |
| --- | --- | --- | --- | --- | --- | --- | --- | --- |
| N. Alberta - Lethbridge | Early | 4.55 | 2.993 | 91 | -2.581 | 11.68 | 1.52 | 0.2863 |
| PEI - Lethbridge | Early | -13.36 | 2.337 | 91 | -18.93 | -7.792 | -5.717 | 4.081e-07 |
| PEI - N. Alberta | Early | -17.91 | 3.077 | 91 | -25.24 | -10.58 | -5.822 | 2.589e-07 |
| N. Alberta - Lethbridge | Late | -7.607 | 1.164 | 91 | -10.38 | -4.834 | -6.536 | 1.12e-08 |
| PEI - Lethbridge | Late | 4.812 | 2.881 | 91 | -2.053 | 11.68 | 1.67 | 0.2222 |
| PEI - N. Alberta | Late | 12.42 | 2.696 | 91 | 5.996 | 18.84 | 4.607 | 3.92e-05 |
| N. Alberta - Lethbridge | Mid | 21.34 | 2.807 | 91 | 14.65 | 28.03 | 7.603 | 5.395e-10 |
| PEI - Lethbridge | Mid | 1.628 | 1.882 | 91 | -2.856 | 6.112 | 0.8652 | 0.6635 |
| PEI - N. Alberta | Mid | -19.71 | 3.15 | 91 | -27.22 | -12.21 | -6.258 | 3.808e-08 |

```
## R version 4.2.2 (2022-10-31 ucrt)
## Platform: x86_64-w64-mingw32/x64 (64-bit)
## Running under: Windows 10 x64 (build 19044)
## 
## Matrix products: default
## 
## locale:
## [1] LC_COLLATE=English_United States.utf8 
## [2] LC_CTYPE=English_United States.utf8   
## [3] LC_MONETARY=English_United States.utf8
## [4] LC_NUMERIC=C                          
## [5] LC_TIME=English_United States.utf8    
## 
## attached base packages:
## [1] stats     graphics  grDevices utils     datasets  methods   base     
## 
## other attached packages:
## [1] car_3.1-1      carData_3.0-5  survival_3.4-0 emmeans_1.8.2  nlme_3.1-160  
## [6] pander_0.6.5   dplyr_1.0.10  
## 
## loaded via a namespace (and not attached):
##  [1] Rcpp_1.0.9         highr_0.9          pillar_1.8.1       bslib_0.4.1       
##  [5] compiler_4.2.2     jquerylib_0.1.4    tools_4.2.2        digest_0.6.30     
##  [9] gtable_0.3.1       jsonlite_1.8.3     evaluate_0.18      lifecycle_1.0.3   
## [13] tibble_3.1.8       lattice_0.20-45    pkgconfig_2.0.3    rlang_1.0.6       
## [17] Matrix_1.5-3       cli_3.4.1          yaml_2.3.6         mvtnorm_1.1-3     
## [21] xfun_0.34          fastmap_1.1.0      withr_2.5.0        stringr_1.4.1     
## [25] knitr_1.40         generics_0.1.3     vctrs_0.5.0        sass_0.4.2        
## [29] grid_4.2.2         tidyselect_1.2.0   glue_1.6.2         R6_2.5.1          
## [33] fansi_1.0.3        rmarkdown_2.18     farver_2.1.1       ggplot2_3.4.0     
## [37] magrittr_2.0.3     scales_1.2.1       htmltools_0.5.3    splines_4.2.2     
## [41] abind_1.4-5        colorspace_2.0-3   xtable_1.8-4       labeling_0.4.2    
## [45] utf8_1.2.2         stringi_1.7.8      estimability_1.4.1 munsell_0.5.0     
## [49] cachem_1.0.6
```
