# Supplementary figures and images for: The effects of protein supplementation, fumagillin treatment, and colony management on the productivity and long-term survival of honey bee (Apis mellifera) colonies
Source: PLoS One. 2024 Mar 15;19(3):e0288953. doi: 10.1371/journal.pone.0288953 (PMC10942092; doi:10.1371/journal.pone.0288953)

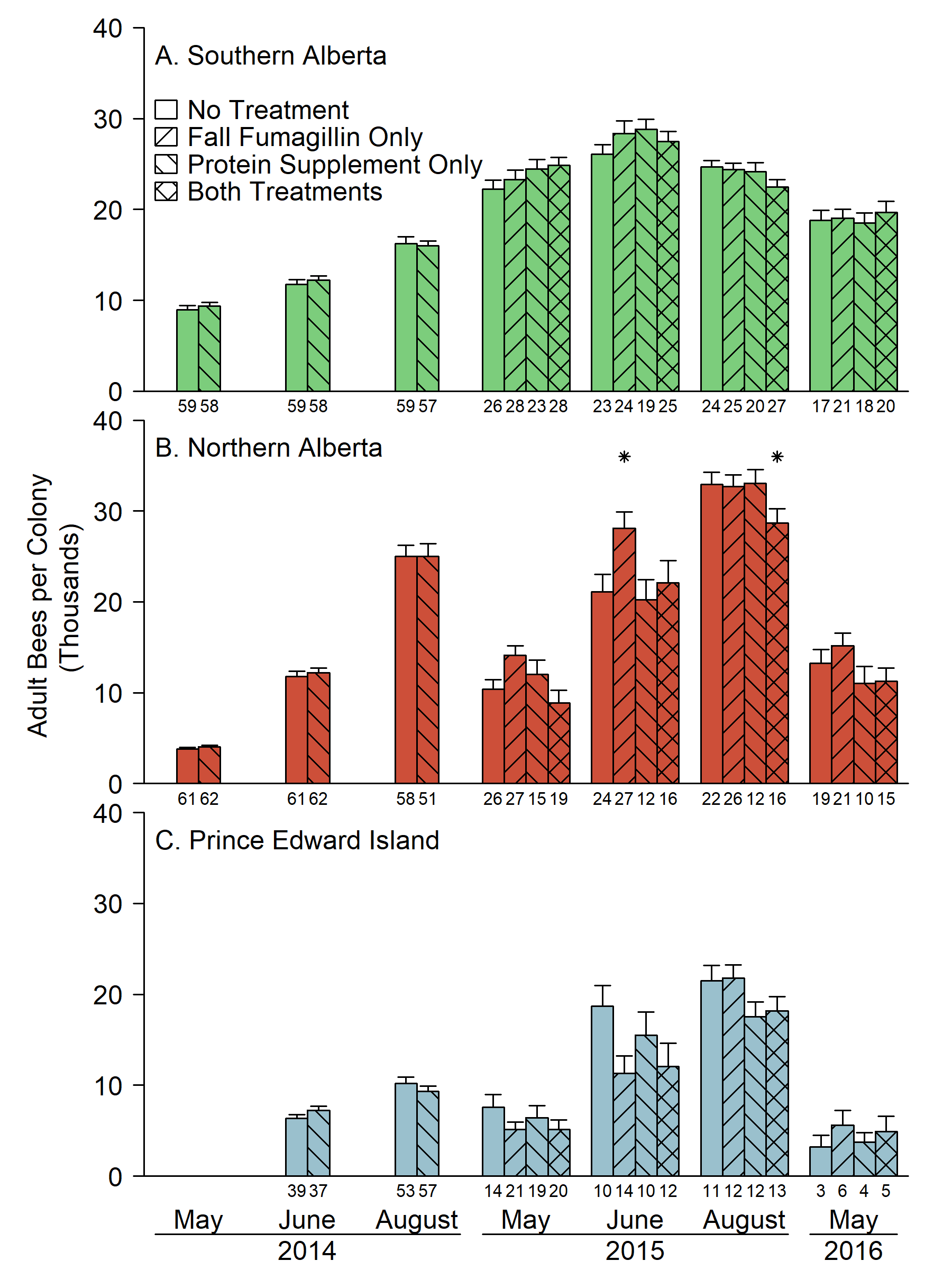

Supplement: S2 Fig — Stars (*) above a column indicate that the treatment group was significantly different from the untreated control group in contrasts within region and date (p<0.05, Bonferroni adjusted; see S5 File). Numbers below a column indicate the number of viable colonies in the treatment group. Fumagillin was first applied in the fall of 2014; consequently, only two columns (untreated and protein supplemented) are shown on the earlier dates; and these columns include colonies subsequently treated with fumagillin. (TIFF) [file pone.0288953.s007.tiff]

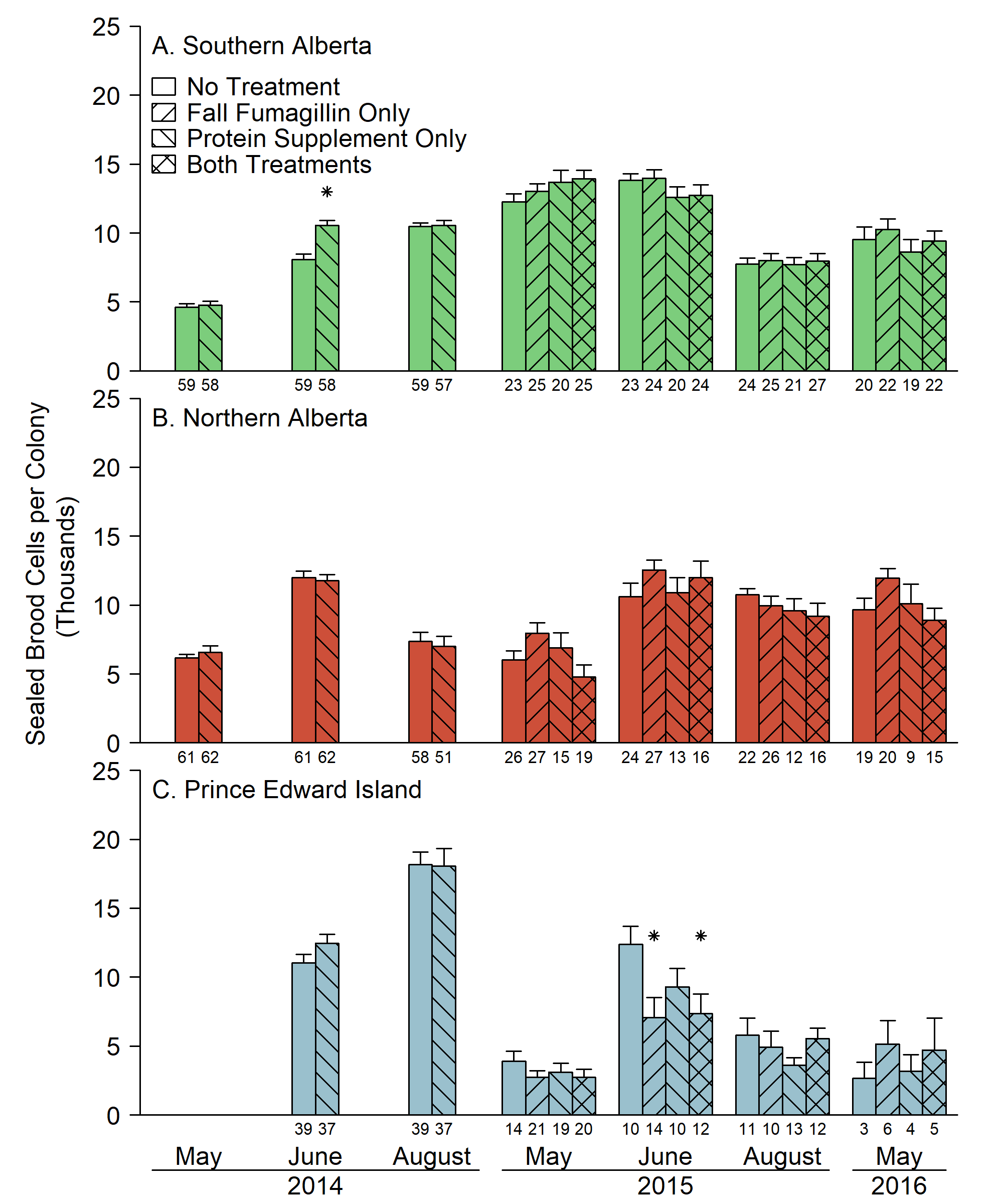

Supplement: S3 Fig — Stars (*) above a column indicate that the treatment group was significantly different from the untreated control group in contrasts within region and date (p<0.05, Bonferroni adjusted; see S5 File). Numbers below a column indicate the number of viable colonies in the treatment group. Fumagillin was first applied in the fall of 2014; consequently, only two columns (untreated and protein supplemented) are shown on the earlier dates; and these columns include colonies subsequently treated with fumagillin. Data shown for PEI in August 2014 reflect parent colonies only; daughter colonies had no sealed brood. (TIFF) [file pone.0288953.s008.tiff]

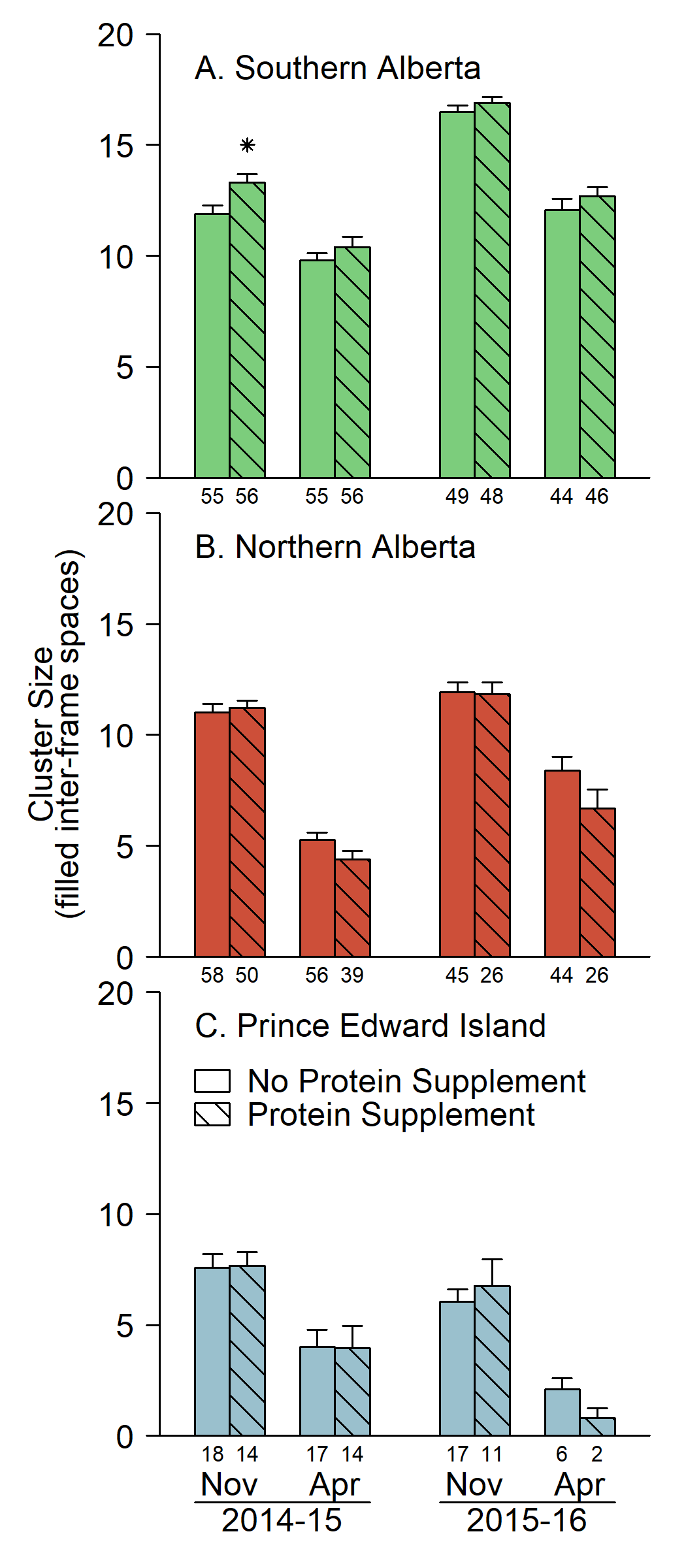

Supplement: S4 Fig — Stars (*) above a column indicate that the protein supplemented group was significantly different from the unsupplemented group in contrasts within region and date (p<0.05, Bonferroni adjusted; see S5 File). Numbers below a column indicate the number of viable colonies in the treatment group. Fumagillin did not affect cluster sizes; therefore, columns include both fumagillin treated and untreated colonies. (TIFF) [file pone.0288953.s009.tiff]

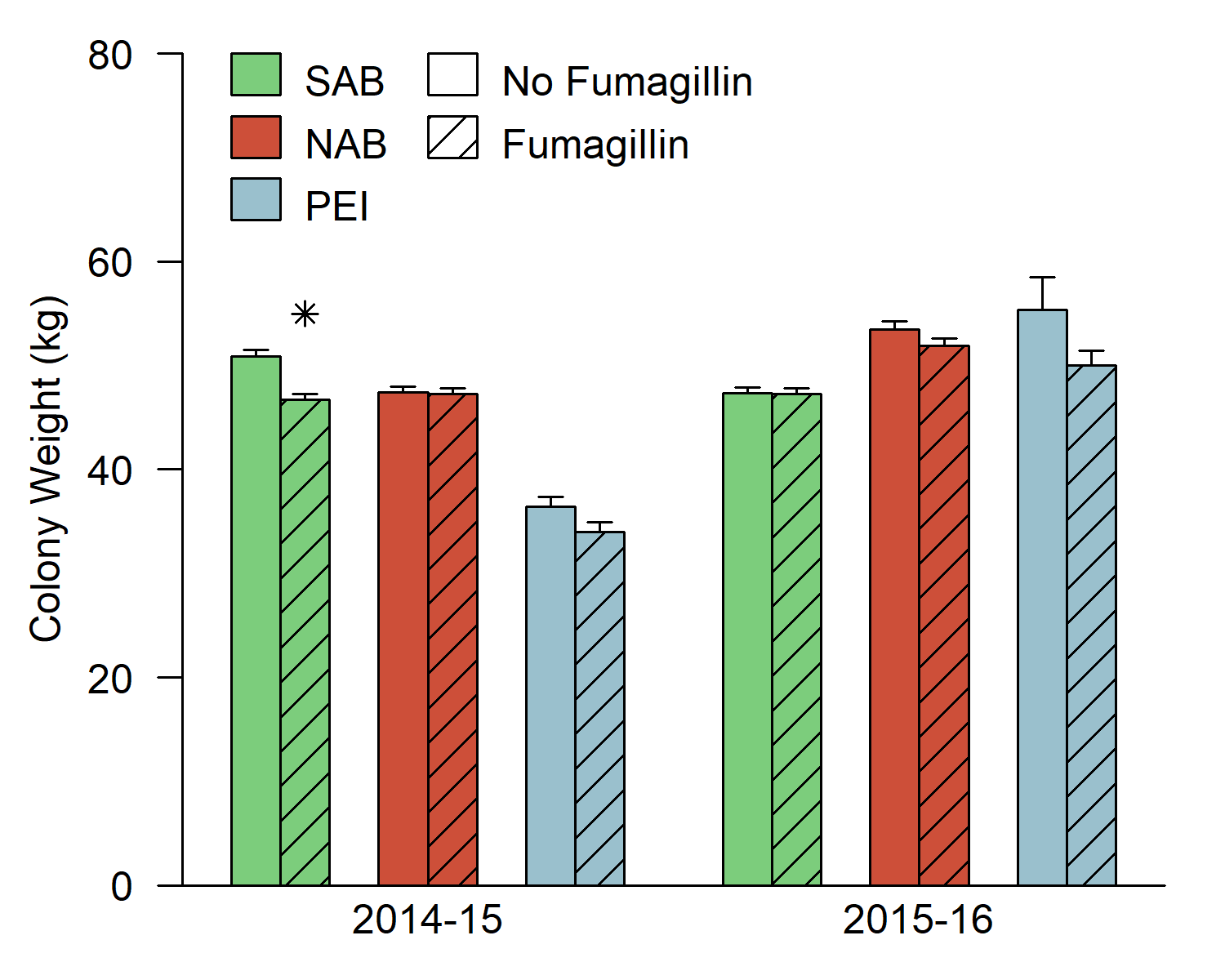

Supplement: S5 Fig — Estimated marginal means are averaged across levels of protein treatment and month because there was no significant interaction between fumagillin and these factors. Each combination of region and year is shown separately because there was a significant three-way interaction of fumagillin, region, and year. Stars (*) above a column indicate that the fumagillin-treated group was statistically different from the corresponding untreated group (p<0.05, Bonferroni adjusted; see S5 File). SAB:Southern Alberta; NAB: Northern Alberta; PEI: Prince Edward Island. (TIFF) [file pone.0288953.s010.tiff]

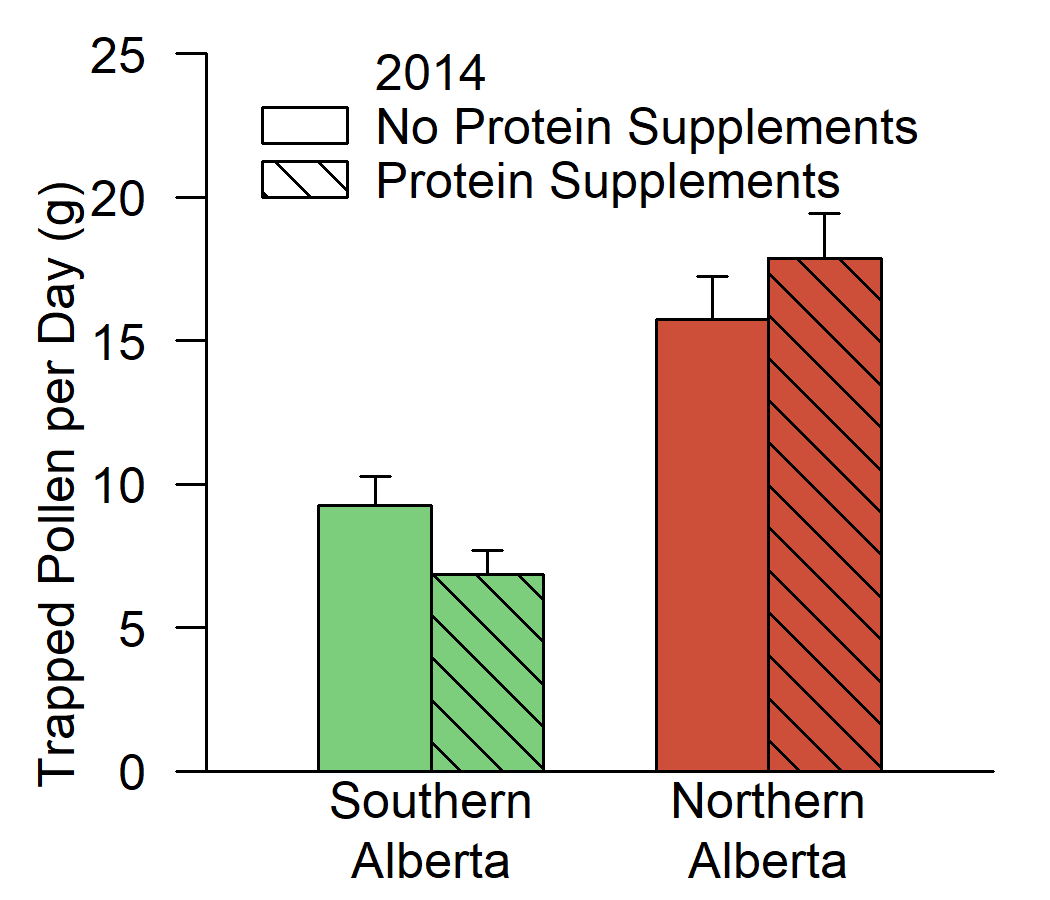

Supplement: S6 Fig — Pollen was trapped for periods of two to four days at intervals during the canola bloom; that is, between the first week of July and early August. (TIFF) [file pone.0288953.s011.tiff]

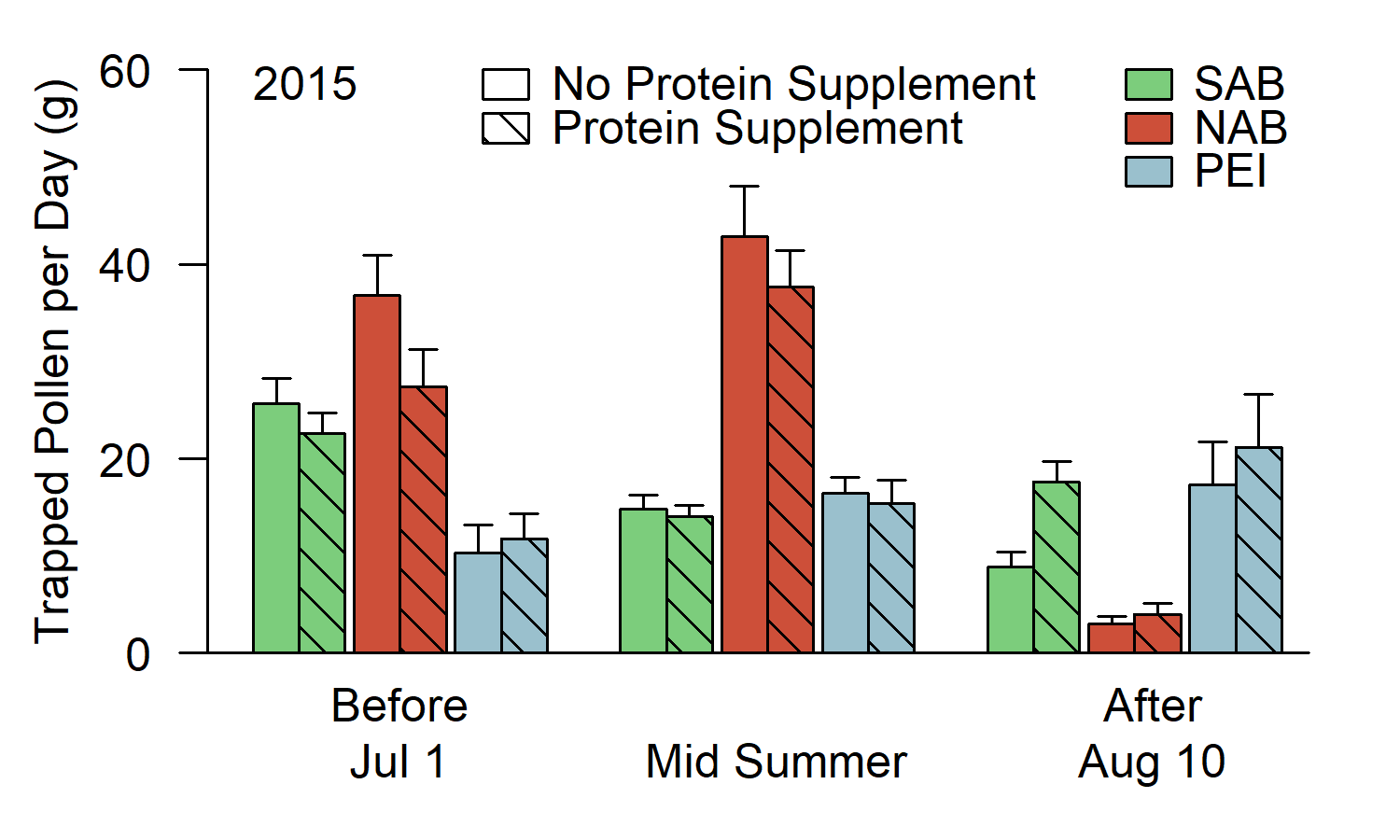

Supplement: S7 Fig — Pollen was trapped from a subset of the largest colonies in each apiary and treatment group, at two-week intervals between mid May and mid September. SAB: Southern Alberta; NAB: Northern Alberta; PEI: Prince Edward Island. (TIFF) [file pone.0288953.s012.tiff]
