# Supplementary material for: The effects of protein supplementation, fumagillin treatment, and colony management on the productivity and long-term survival of honey bee (Apis mellifera) colonies
Source: PLoS One. 2024 Mar 15;19(3):e0288953. doi: 10.1371/journal.pone.0288953 (PMC10942092; doi:10.1371/journal.pone.0288953)
Supplement: Supplementary file 8 [file pone.0288953.s001.odt]

## Colony establishment and management

The colonies were established in late April 2014 by splitting local overwintered hives (SAB) or hiving 1 kg packages of bees imported from New Zealand (NAB).  In PEI, colonies were established from splits made at blueberry pollination sites in mid-June. The target size for splits at both locations was 8 frames of bees with 4 or 5 frames of brood. The standard management practices of the PEI beekeeper also included splitting colonies in late summer, and as such daughter colonies were also included in the study. Daughter colonies were always assigned to the same treatment group as the parent colony. In each region, newly mated Carniolan queens (Kona Queen Company, HI, USA) were introduced to each colony established at the beginning of the study. Queens were marked with green paint on their thorax, and their wings were clipped to prevent swarming and aid in their identification. Both spontaneous and beekeeper-initiated re-queening were permitted, and new queens were marked and recorded when observed by project staff. In the case of re-queening by beekeepers, and of the late summer splits in PEI, local stock was used.

Beekeepers used their own equipment and management procedures. All producers exclusively used Langstroth deep boxes. Colonies were in groups of four per pallet and were wintered outdoors in two Langstroth deep boxes. Both Alberta producers used an insulating wrap and cover on each pallet. The PEI beekeeper used only an insulating top cover. No producer used antibiotics for the control of brood diseases. All producers controlled Varroa destructor using Apivar® (amitraz) strips during the spring and fall (Alberta locations) or used Apivar® in summer and oxalic acid in winter (PEI). 

There were two or three apiary-level groups per region (Table S1), each with approximately 40 colonies, for initial totals per region of 117 (SAB), 123 (NAB) and 76 (PEI) colonies. All producers moved hives locally multiple times as part of normal management practice (Table S1), but during the bloom period of the principal crop (lowbush blueberries or canola), most PEI and all Alberta colonies were either within, or immediately next to, fields of that crop.

Colony assessment procedures

All adult bee assessments began at first light and ended with the start of bee flight in the morning, to ensure the entire hive population was measured. Sealed brood assessments were usually done later the same day. 

Visual assessments

For each frame within a hive, the area covered by bees or by sealed brood was estimated as described by Delaplane et al. [33]. Each measurement was performed by a single inspector. The inspector laid a grid of 1-inch (2.54 cm) squares over the frame and counted the number of squares filled with adult bees or sealed brood. The values from each frame were summed to yield brood and bee areas for the colony.

Digital images

Methods for counting bees with digital images have been previously described [32]. A specially constructed frame stand (Fig S1) with an opaque back and translucent sides was used to ensure uniform light in the image. A Nikon D7100 camera with a Nikkor 105 mm Micro VR lens was set on a tripod approximately 2 m from the position of the frames, such that the edges of the frames would be just inside the boundaries of the image. Each side of each frame with bees or sealed brood was photographed. Frames were handled as quickly and gently as possible, working from the top of the hive to the bottom with minimal smoke.  Unphotographed bees (bees on the box, bottom board, etc.) were visually estimated by area to the nearest equivalent of a quarter frame side; for this purpose, one side of a frame was assumed to hold 1200 bees [35].

Cluster size measurements

For population assessments in late fall and early spring, we considered the temperature too cold to break the cluster and examine hives frame by frame. Instead, we estimated cluster size (Fig S1, below), which is the number of inter-frame spaces filled with bees to the nearest ¼ of a space, as viewed from above and below [36]. Observations from the top and bottom of each box were averaged to give the cluster score for the box; the value for the hive was the sum of the scores for the boxes.

Queen and disease inspections, and sample collection

Inspections for queen and disease status, and samples of bees were either done the same day as the population size assessments, or the following day. Frames of bees were gently removed from the hive and examined in turn until either the queen had been found or all frames had been examined twice. While looking for the queen, inspectors noted any unusual looking adult bees – deformities, abnormal behaviours, or external parasites as well as any brood with signs of disease. Once the queen was found, the frame she was on was set gently outside the hive, and two samples of bees were taken from the remaining frames: 1/2 sample vial (~150 bees) from a frame with honey and no brood, and 1 full sample vial (~ 400 bees) from a frame with brood. In November and April, colonies in PEI were not inspected, and colonies in Alberta were inspected rapidly to avoid chilling bees and brood (pest and pathogen measurements from these samples will be the subject of upcoming manuscripts).

Weight measurements and pollen traps

Weight measurements used digital platform scales (model GBK 260A, Adam Equipment, Oxford, CT, USA or equivalent), or a hanging scale (Optima digital crane scale, Model WGB1483661, Global Industrial Canada, Toronto, ON, Canada).

We measured the effects of treatment and region on the quantity of pollen collected. In 2014, each research location used the pollen traps that were available locally, which for Southern Alberta were front entrance style traps (Propolis-etc, St. Mathieu de Beloeil, QC, Canada) and for Northern Alberta were Ontario Agricultural College-style traps [37], previously custom built.  In 2015, all three locations used the front entrance style traps.

Estimating numbers of bees and brood

... from visual assessments

Area measurements from visual assessments were converted to estimated numbers of bees or brood using the following conversion factors: 8.70 adult bees and 25.3 sealed brood cells per square inch (1.35 bees and 3.93 sealed brood cells per cm2). The conversion factors were estimated from 33 images of adult bees and 12 images of sealed brood taken in 2015 (Tables S6 and S7).

... from photographic assessments

Images of adult bees or sealed brood for each colony were imported to an image analysis program, Honeybee Complete® 4.2 (WSC Scientific GmbH, Heidleberg, Germany). This software automatically identifies, labels, and counts each identified bee or sealed brood cell in images, but with some inherent errors. Each analyzed image was screened, and if necessary corrected, by a human observer. In the case of sealed brood, results were often close to 100% accuracy, and no adjustment was applied unless the number of false detections or failed detections in the image appeared to exceed 3% of the total (observer's visual estimate).

Three methods of correction were used, as needed: 1) If individual bees or brood were concentrated in one part of the image, the area selection tool of the software was used to exclude other parts of the image from analysis;  2) For individual bee recognition errors, an  adjustment was applied by manually selecting or deselecting using the software; or 3) For adult bee estimates, correction factors were applied to account for HoneyBee Complete’s systematic under-recognition of the true number of individuals in an image. Correction factors were calculated for images of typical quality in each combination of region and date (Table S8). Images of poorer quality were assigned a custom correction factor or were manually counted.

Statistical details

Cox proportional hazards models

Colony survival and queen survival were analyzed as Cox proportional hazards models, using the R Survival package, version 3.3-1 [40, 41]. Hazards were not proportional over time among regions; consequently, region was a stratified factor in the model. The split colonies at PEI could not be regarded as independent of their parents, so related colonies were grouped using a cluster command. Since fumagillin was not applied until the end of the first field season, some deaths among colonies assigned to the fumagillin treated group could not be attributed to fumagillin. To resolve this, the dataset was split into non-overlapping time periods for each colony as described by Therneau et al. [42].

Mixed models

Measures of colony population (adult bee counts, sealed brood counts, and cluster sizes), productivity (honey production, pollen collection), and syrup feed consumption (colony weight) were analyzed as dependent variables in linear mixed effects models using the R nlme package, version 3.1_153. For a comparison of linear mixed effects models with other approaches to handling repeated measures data, see Ma et al. [43]. Most dependent variables were modelled without transformation and with variances weighted by date or by date and region to reduce heteroskedasticity. The independent (fixed effects) variables under consideration were region, date, protein supplement treatment, and fumagillin treatment, and their interactions. Random intercepts and random slopes by colony, as well as several correlation structures were compared and the models with the best fit statistics were selected (S4 and S5 Files). Final models were selected by successively removing non-significant higher order interactions until only interactions that were significant at P<0.05 or necessary to represent the experimental structure remained.

Some dependent variables (adult bees, brood, and honey production) were measured repeatedly both before and after the first fumagillin application in fall 2014; consequently, the main effect of "fumagillin" was not meaningful over all time points,  however the interaction of fumagillin with date allowed interpretation of effects and was inherently implied by the project design. Therefore, both were retained in the models even if they were not significant at P<0.05.

Contrasts

Statistical significance (p) values for contrasts are shown without adjustment for multiple comparisons; instead, the significance threshold (p=0.05) was adjusted with a Bonferroni correction (S5 File). When the analysis of variance indicated that a factor and its interaction were both significant, we present contrasts for both. In such cases, the interactions were usually differences in the size of the treatment effect, not the direction, and as such the significant interaction does not exclude the more general result.

Adult bee count and sealed brood count models

There was no data for May 2014 at PEI and the practice there of splitting colonies in late summer complicated the analysis of colony populations. For simplicity, most statistics in the paper refer to colony sizes as observed (without adjusting for the effects of splitting). To clarify the influence of splitting, each statistical model was also tested on variants of the dataset that either excluded all split colonies, excluded colonies that were not split, or attributed all productivity of each daughter colony to the parent colony. These analyses are discussed only where the results differed from the main analysis. Because date was treated as a categorical fixed effect and the interaction of date and region was important, the lack of data from PEI in May 2014 meant that May 2014 data from the other sites had to be dropped from analysis. An alternative model was considered which would have allowed the whole dataset to be used by dividing the date term into a seasonal component ("month") and a continuous component ("assessment"), but the results were similar to those presented.

Cluster size and colony weight models

The linear mixed models for cluster size and colony weight data included five fixed effects: "winter" (2014 or 2015), "month" (November or April), "region", "patties", and "fumagillin". Colonies that died during winter (and therefore had no April measurement) were dropped from the November data for the same winter so that comparisons between spring and fall would reflect results from the same colonies. In addition, after a preliminary analysis we found that recently split colonies were smaller than other colonies, and the number of splits was not uniform among treatment groups, which resulted in a region-treatment interaction that was unrelated to the effects of treatment. Alternate models were used to study this problem (analyzing regions separately; analyzing split and non-split colonies separately; assigning all bees from the daughter split to the parent), but in the results presented, colonies split in August were excluded from the dataset for the following winter.

The linear models for the change in cluster size and change in weight during winter included four fixed effects: "winter" (2014 or 2015), "region", "patties", and "fumagillin". We also considered the possibility that apparent treatment-related effects on the change in cluster size and colony weight might be functions of colony size at the beginning of winter. Therefore, cluster size in November, and its interactions, were added as predictors in the models for the change in weight and the change in cluster size. 

Honey and pollen collection models

Honey production was modelled as a linear mixed model with fixed effects "year" (2014 or 2015), "region" (SAB or NAB), "patties", and "fumagillin".

Pollen collection data was square root transformed before analysis to reduce skewness. Different models were used for 2014 and 2015 data because the experimental procedure was different. In 2014, total pollen collected per colony was analyzed using a mixed model with three fixed factors: region (SAB or NAB), protein supplements (yes or no), and pollen collection date.  Since each colony was tested only once, in this case the random factor was "apiary".

In 2015, pollen was trapped repeatedly from a subset of colonies within each region. Fumagillin was not expected to affect pollen collection and was excluded from the model after a preliminary inspection of the data showed no evidence of an effect. The model contained three fixed effects: region (SAB, NAB, PEI), protein supplements (yes or no), and season (before canola bloom, during canola bloom, and after canola bloom). Season was used rather than date because the dates of testing were different for different colonies and sites, and to account for the fact that protein supplements were not applied during the canola bloom. Because repeated measurements were taken, "colony" was included as a random effect.  Variances were weighted by date (not season) and region.
