# Supplementary material for: The effects of protein supplementation, fumagillin treatment, and colony management on the productivity and long-term survival of honey bee (Apis mellifera) colonies
Source: PLoS One. 2024 Mar 15;19(3):e0288953. doi: 10.1371/journal.pone.0288953 (PMC10942092; doi:10.1371/journal.pone.0288953)
Supplement: Supplementary file 9 [file pone.0288953.s013.odt]

S1 Table. Apiary-level groups of honey bee colonies, their locations, and dates of movement

| Group1 | Colonies | Period | Dates (DD-MM-YYYY)2 | Latitude2 | Longitude2 |
| --- | --- | --- | --- | --- | --- |
| SAB1 | 1-40 | Spring, 2014 | 01-05-2014 to 21-07-2014 | 49.726380 | -112.656428 |
|  |  | Canola Bloom 2014 | 21-07-2014 to 20-08-2014 | 49.859367 | -111.912900 |
|  |  | Winter 2014-15 | 20-08-2014 to 27-06-2015 | 49.726380 | -112.656428 |
|  |  | Canola Bloom 2015 | 27-06-2015 to 27-07-2015 | 49.929966 | -112.679866 |
|  |  | Winter 2015-16 | 27-07-2015 to 05-05-2016 | 49.821315 | -112.666238 |
| SAB2 | 41-80 | Spring, 2014 | 01-05-2014 to 08-07-2014 | 49.821315 | -112.666238 |
|  |  | Canola Bloom 2014 | 08-07-2014 to 09-08-2014 | 49.844497 | -111.809746 |
|  |  | Winter 2014-15 | 09-08-2014 to 23-06-2015 | 49.821315 | -112.666238 |
|  |  | Canola Bloom 2015 | 23-06-2015 to 08-08-2015 | 49.746538 | -112.582464 |
|  |  | Winter 2015-16 | 08-08-2015 to 05-05-2016 | 49.726380 | -112.656428 |
| SAB3 | 81-120 | Spring, 2014 | 01-05-2014 to 11-07-2014 | 49.760569 | -112.420968 |
|  |  | Canola Bloom 2014 | 11-07-2014 to 09-08-2014 | 49.930782 | -111.700032 |
|  |  | Winter 2014-15 | 09-08-2014 to 01-07-2015 | 49.760569 | -112.420968 |
|  |  | Canola Bloom 2015 | 01-07-2015 to 06-08-2015 | 49.882954 | -112.552232 |
|  |  | Winter 2015-16 | 06-08-2015 to 05-05-2016 | 49.760569 | -112.420968 |
| NAB1 | 201-240 | Spring 2014 | 24-04-2014 to 09-07-2014 | 55.203245 | -119.396083 |
|  |  | Canola Bloom 2014 | 09-07-2014 to 17-09-2014 | 55.28781 | -119.14670 |
|  |  | Winter 2014-15 | 17-09-2014 to 06-07-2015 | 55.197581 | -119.300508 |
|  |  | Canola Bloom 2015 | 06-07-2015 to 10-09-2015 | 55.300545 | -119.282378 |
|  |  | Winter 2015-16 | 10-09-2015 to 06-05-2016 | 55.197581 | -119.300508 |
| NAB2 | 241-280 | Spring 2014 | 24-04-2014 to 09-07-2014 | 55.203245 | -119.396083 |
|  |  | Canola Bloom 2014 | 09-07-2014 to 16-09-2014 | 55.28773 | -119.27303 |
|  |  | Winter 2014-15 | 17-09-2014 to   04-2015 | 55.197581 | -119.300508 |
|  |  | Spring 2015 | 04-2015 to 08-07-2015 | 55.310554 | -119.306549 |
|  |  | Canola Bloom 2015 | 08-07-2015 to 10-09-2015 | 55.330756 | -119.254856 |
|  |  | Winter 2015-16 | 10-09-2015 to 06-05-2016 | 55.197581 | -119.300508 |
| NAB3 | 281-325 | Spring 2014 | 24-04-2014 to 10-07-2014 | 55.185800 | -119.298185 |
|  |  | Canola Bloom 2014 | 10-07-2014 to 16-09-2014 | 55.337567 | -119.258650 |
|  |  | Winter 2014-15 | 17-09-2014 to 06-07-2015 | 55.197581 | -119.300508 |
|  |  | Canola Bloom 2015 | 07-07-2015 to 10-09-2015 | 55.279759 | -119.308138 |
|  |  | Winter 2015-16 | 10-09-2015 to 06-05-2016 | 55.197581 | -119.300508 |
| PEI1a | 401-424, 447-454 | 2014 | 02-07-2014 to 10-12-2014 | 46.273895 | -62.757895 |
|  | 2015 | 21-05-2015 to 24-05-2016 | 46.418846 | -62.770763 |
| PEI1b | 455-465, 481-507 | June 2014 | 03-07-2014 | 46.308457 | -62.744076 |
|  | All Other Dates | 21-08-2014 to 24-05-2016 | 46.418846 | -62.770763 |
| PEI2a | 436-441, 468-480, 512-519 | June 2014 | 25-06-2014 | 46.308457 | -62.744076 |
|  | All Other Dates | 20-08-2014 to 24-05-2016 | 46.429000 | -62.769577 |
| PEI2b | Splits | Fall 2014 | 30-08-2014 to 10-12-2014 | 46.304864 | -63.058812 |
|  | 2015 | 22-05-2015 to 24-05-2016 | 46.429000 | -62.769577 |

1Group descriptors: SAB: Southern Alberta; NAB: Northern Alberta; PEI: Prince Edward Island.

2For Alberta locations, dates and coordinates are exact. For PEI locations, the beekeeper did not keep precise movement records; dates are as observed at inspection, coordinates are approximate, and grouped colonies were  occasionally separated, particularly during blueberry bloom (June inspections).
