# Supplementary material for: The effects of protein supplementation, fumagillin treatment, and colony management on the productivity and long-term survival of honey bee (Apis mellifera) colonies
Source: PLoS One. 2024 Mar 15;19(3):e0288953. doi: 10.1371/journal.pone.0288953 (PMC10942092; doi:10.1371/journal.pone.0288953)
Supplement: Supplementary file 10 [file pone.0288953.s014.odt]

S2 Table. Replicates by location and treatment

| Apiary Group | Number of Colonies |
| --- | --- |
|  | No Protein Supplement | Protein Supplement |
|  | No Fumagillin | Fumagillin | No Fumagillin | Fumagillin |
| SAB1 | 10 | 9 | 10 | 10 |
| SAB2 | 10 | 10 | 9 | 10 |
| SAB3 | 10 | 10 | 9 | 10 |
| NAB1 | 10 | 10 | 10 | 10 |
| NAB2 | 10 | 10 | 10 | 10 |
| NAB3 | 11 | 10 | 11 | 11 |
| PEI1a | 7 | 7 | 8 | 7 |
| PEI1b | 4 | 9 | 5 | 7 |
| PEI2a - Original Colonies2 | 7 | 5 | 5 | 5 |
| PEI2b-2014 Splits | 6 | 8 | 7 | 13 |
| PEI2b-2015 Splits | 3 | 1 | 5 | 3 |

1Group descriptors: SAB: Southern Alberta; NAB; Northern Alberta; PEI: Prince Edward Island

2Original Colonies denotes colonies at this location that had a start date of June 2014. "Splits" denotes colonies started in late summer from colonies in either the PEI1 or PEI2 groups; the parent colony (original queen and box) remained at the original location. All daughter colonies (new queen and box) were subsequently managed with the colonies from group PEI2. Since the splits were produced by the beekeeper based on the perceived strength of the parent colonies, the number of splits in each treatment group is an outcome, rather than a design element of the experiment.
