# Supplementary material for: The effects of protein supplementation, fumagillin treatment, and colony management on the productivity and long-term survival of honey bee (Apis mellifera) colonies
Source: PLoS One. 2024 Mar 15;19(3):e0288953. doi: 10.1371/journal.pone.0288953 (PMC10942092; doi:10.1371/journal.pone.0288953)
Supplement: Supplementary file 11 [file pone.0288953.s015.odt]

S3 Table. The protein supplement treatment: quantities and dates of supplement application1

| Apiaries2 | Period3 | First Application (DD-MM-YYYY) | Last Application (DD-MM-YYYY) | Quantity (total number of 454g patties applied to each colony during period) |
| --- | --- | --- | --- | --- |
| Southern Alberta (all sites)4 | Pre-Bloom 2014 | 21-05-2014 | 10-06-2014 | 5 |
| Post-Bloom 2014 | 15-09-2014 | 22-10-2014 | 7 |
| Pre-Bloom 2015 | 01-05-2015 | 04-06-2015 | 8 |
| SAB1 | Post-Bloom 2015 | 09-09-2015 | 17-10-2015 | 8 |
| SAB2 | Post-Bloom 2015 | 29-08-2015 | 17-10-2015 | 6.25 |
| SAB3 | Post-Bloom 2015 | 29-08-2015 | 17-10-2015 | 7.75 |
|  |  |  |  |  |
| Northern Alberta (all sites)4 | Pre-Bloom 2014 | 20-05-2014 | 02-07-2014 | 4 |
| Post-Bloom 2014 | 21-08-2014 | 30-10-2014 | 6 |
| Pre-Bloom 2015 | 11-05-2015 | 06-07-2015 | 5 |
| Post-Bloom 2015 | 06-08-2015 | 30-10-2015 | 6 |
|  |  |  |  |  |
| Prince Edward Island (all sites)4 | 2014 | 15-06-2014 | 10-12-2014 | ad libitum5 |
| 2015 | 25-05-2015 | 12-12-2015 | ad libitum |

1 Patties with 25% pollen were applied (only to the protein supplemented group) by the local commercial beekeeper, approximately every two weeks.

2Apiaries refers to the apiary level groupings of colonies, as described in Table 1, above.

3 Pre-Bloom and Post-Bloom refer to the bloom period of canola (Brassica napus L.). In Alberta, supplements were not applied during the bloom period.

4 Specific application practices varied among beekeepers, partly because consumption rates varied. Consumption was generally highest in Southern Alberta and lowest in Prince Edward Island. The Southern Alberta beekeeper occasionally  supplied more than one patty at a time per colony. The Northern Alberta colonies received 1 patty every two weeks. The Prince Edward Island colonies received a new patty once the previous patty had been consumed.

5 In Prince Edward Island, the quantity applied varied by colony needs and was not recorded by the beekeeper.
