# Supplementary material for: The effects of protein supplementation, fumagillin treatment, and colony management on the productivity and long-term survival of honey bee (Apis mellifera) colonies
Source: PLoS One. 2024 Mar 15;19(3):e0288953. doi: 10.1371/journal.pone.0288953 (PMC10942092; doi:10.1371/journal.pone.0288953)
Supplement: Supplementary file 12 [file pone.0288953.s016.odt]

S4 Table. Fall fumagillin treatments by region

| Region | Treatment Start | Method | Detail |
| --- | --- | --- | --- |
| SAB | 15-09-2014 | Feed +  Drench | Feed: 100 mg fumagillin in 5 liters syrup1 Drench: 3 x 33.3 mg in 250 ml syrup. |
| 29-09-2015 | Drench | Drench: 4 x 50 mg in 250 ml syrup |
| NAB | 18-09-2014 | Feed | 200 mg fumagillin in 8 liters syrup. |
| 12-09-2015 | Feed | 200 mg fumagillin in 8 liters syrup. |
| PEI | 20-10-2014 | Feed | 200 mg fumagillin in 8 liters syrup. |
| 10-2015 | Feed | 200 mg fumagillin in 8 liters syrup. |

1 The syrup was a 67 Brix solution of sucrose in water, in all cases.
