# Supplementary material for: The effects of protein supplementation, fumagillin treatment, and colony management on the productivity and long-term survival of honey bee (Apis mellifera) colonies
Source: PLoS One. 2024 Mar 15;19(3):e0288953. doi: 10.1371/journal.pone.0288953 (PMC10942092; doi:10.1371/journal.pone.0288953)
Supplement: Supplementary file 13 [file pone.0288953.s017.odt]

S5 Table. Colony assessment dates

| Nominal Date | Assessment Type | Exact Dates, by Region |
| --- | --- | --- |
|  |  |  | SAB | NAB | PEI |
| 2014 | May | Grid Area | 21-23 May | 20-21 May | n/a |
|  | Jun | Grid Area | 23-27 Jun | 24-26 Jun | 25 Jun, 2-4 Jul |
|  | Aug | Grid Area | 18-22 Aug | 14-15, 18-21 Aug | 20-22, 30 Aug1 |
|  | Nov | Cluster Area | 29 Oct | 21-23 Oct | 1-12 Dec2 |
| 2015 | Apr | Cluster Area | 26 Mar | 10 Apr | 23 May3 |
|  | May | Photographic | 12-14 May | 11-13 May | 21-22 May |
|  | Jun | Photographic | 22-24 Jun | 23-26 Jun | 6-9 Jul |
|  | Aug | Photographic | 1,3,8 Sep | 18-27 Aug | 21-24 Sep |
|  | Nov | Cluster Area | 21 Oct | 23 Oct4 | 12 Dec |
| 2016 | Apr | Cluster Area | 30 Mar | 30 Mar4 | 7 May |
|  | May | Photographic | 3-5 May | 5-6 May | 24 May |

1 Original colonies 20-22 Aug; splits 30 Aug.

2 Date estimated.

3 April 2015 cluster and weight data at PEI were collected after the photographic assessments because of cold spring weather and a shortage of trained staff in PEI.

4 Date of cluster measurements; colony weights were measured on 16 October 2015 & 27 April 2016.
