# Supplementary material for: The effects of protein supplementation, fumagillin treatment, and colony management on the productivity and long-term survival of honey bee (Apis mellifera) colonies
Source: PLoS One. 2024 Mar 15;19(3):e0288953. doi: 10.1371/journal.pone.0288953 (PMC10942092; doi:10.1371/journal.pone.0288953)
Supplement: Supplementary file 15 [file pone.0288953.s019.odt]

S7 Table. Dataset used to estimate the relationship between area measurements and counts of sealed worker brood cells1

| Sealed Brood Cell Count2 | Area Covered by Sealed Brood (Proportion of Frame)3 | Area Covered by Sealed Brood (cm2)4 | Area per Brood Cell (cm2)5 |
| --- | --- | --- | --- |
| 211 | 0.0550 | 57.6675 | 0.273305687 |
| 295 | 0.0714 | 74.8629 | 0.253772542 |
| 437 | 0.1081 | 113.34285 | 0.259365789 |
| 440 | 0.1007 | 105.58395 | 0.239963523 |
| 449 | 0.1038 | 108.8343 | 0.24239265 |
| 533 | 0.1305 | 136.82925 | 0.256715291 |
| 775 | 0.1764 | 184.9554 | 0.238652129 |
| 1029 | 0.2510 | 263.1735 | 0.25575656 |
| 1149 | 0.2800 | 293.58 | 0.255509138 |
| 1504 | 0.3550 | 372.2175 | 0.24748504 |
| 1789 | 0.4539 | 475.91415 | 0.266022443 |
| 1853 | 0.4696 | 492.3756 | 0.265718079 |

1The digital images were selected from images collected in this project in May and June 2015.

2 Determined as described using the HoneyBee Complete® 4.2 software

3 Using the area selection tool of the HoneyBee Complete® 4.2 software, a polygon corresponding to the perimeter of the photographed frame was drawn. A second polygon was drawn tightly around the sealed worker brood, and its area was expressed as a proportion of the area of the first polygon. The second polygon was positioned so that any area covered by sealed brood outside the polygon was offset by areas inside the polygon uncovered by  brood. 

4 Proportion of Frame * Area of Langstroth Frame (exterior dimensions of 45.0 and 23.3 cm; 1048.5 cm2)

5 Area per sealed worker brood cell (mean ± SD) : 0.255 ± 0.011 cm2; sealed brood cells per cm2 = 1/mean area per brood cell =  3.93.
