# Supplementary material for: The effects of protein supplementation, fumagillin treatment, and colony management on the productivity and long-term survival of honey bee (Apis mellifera) colonies
Source: PLoS One. 2024 Mar 15;19(3):e0288953. doi: 10.1371/journal.pone.0288953 (PMC10942092; doi:10.1371/journal.pone.0288953)
Supplement: Supplementary file 16 [file pone.0288953.s020.odt]

S8 Table. Details of photographic image analysis of adult bees using the Honeybee Complete® 4.2 software1

| Date | Region | Number of Images by Quality Category2 | "Typical" Images |
| --- | --- | --- | --- |
|  |  | Typical | Poor | Manual | Detection Percentage3 | Correction Factor3,4 |
| May 2015 | SAB | 3291 | 245 | 26 | 63.4 | 1.631475073 |
|  | NAB | 2010 | 133 | 86 | 58.8 | 1.755227210 |
|  | PEI | 1046 | 17 | 36 | 70.5 | 1.452273178 |
| June 2015 | SAB Team 1 | 1446 | 120 | 14 | 68.1 | 1.533825193 |
|  | SAB Team 2 | 1932 | 10 | 25 | 67.8 | 1.538082066 |
|  | NAB | 2772 | 62 | 28 | 57.0 | 1.836959152 |
|  | PEI | 1138 | 0 | 2 | 76.3 | 1.326112022 |
| Aug 2015 | SAB Team 1 | 817 | 5 | 0 | 54.6 | 1.929923175 |
|  | SAB Team 2 | 2444 | 2 | 1 | 63.7 | 1.628312211 |
|  | NAB | 3841 | 147 | 21 | 56.0 | 1.909417537 |
|  | PEI | 1571 | 20 | 5 | 62.4 | 1.632301198 |
| May 2016 | SAB Team 1 | 917 | 84 | 42 | 60.7 | 1.711748699 |
|  | SAB Team 2 | 1367 | 0 | 1 | 72.4 | 1.421762268 |
|  | NAB | 1748 | 2 | 18 | 70.4 | 1.485127176 |
|  | PEI | 217 | 3 | 2 | 66.4 | 1.571133005 |

1 As the HoneyBee Complete 4.2 software typically under-estimates, but occasionally overestimates the number of bees in an image,  a screening and categorization procedure was applied to the images both before and after analysis by the software, and correction factors were applied to the output.  Factors associated with overestimates were: areas of bare foundation (undrawn comb), images with light shining through the comb, small numbers (25 or fewer) of bees in the image, and bees in the image but not on the comb. False positives were removed by using the area selection tool to exclude irrelevant parts of the image, or by manual adjustments to the count. Factors associated with unusually large underestimates included out of focus and out of exposure images, light coloured comb, direct sunlight on comb or on the camera lens.  Even with uniformly high quality images, the proportion of bees detected varied, and consequently corrections were applied to the output.

2 Image quality categories: "Typical" means image quality similar to the majority of images taken in the same region and assessment date, in terms of focus, clarity, and exposure. "Poor" means images of a lower quality but sufficient for some bees to be recognized by the software. "Manual" means that the number of bees was counted by a human observer, most often because the image was severely out of focus.

3 Procedure to determine the percent of bees recognized in an individual image, the correction factor to be applied to that image, and the estimated number of bees for the image: 1. Analyze the image with the Honeybee Complete® software and record the number of bees detected. 2. Use the area selection tool to select a cross-section of the image that contains at least 30 detected individual bees, and determine the number of detected bees in the cross section. 3. Manually count the number of bees visible in the cross-section. The recognition percentage is : 100* (detected bees in cross section/ actual bees in cross section); the correction factor is : actual bees in cross section/detected bees in cross section; and the estimated count for the whole image is  detected bees in whole image * correction factor. 

4. Groups of images of similar quality were assigned a common correction factor. Correction factors were determined for twenty five randomly chosen individual images from the group and the mean correction factor was applied to the group. Group correction factors were calculated for typical images from each combination of region and date, and for poor quality images whenever a group of consecutive images all had similar defects.
