# Supplementary material for: The effects of protein supplementation, fumagillin treatment, and colony management on the productivity and long-term survival of honey bee (Apis mellifera) colonies
Source: PLoS One. 2024 Mar 15;19(3):e0288953. doi: 10.1371/journal.pone.0288953 (PMC10942092; doi:10.1371/journal.pone.0288953)
Supplement: Supplementary file 17 [file pone.0288953.s021.odt]

S9 Table. Colony and queen survival to the end of the experiment.1

| Region & treatment | Total colonies | Colony deaths | Surviving colonies3 | Surviving original queens3 | Successful queen replacements4 |
| --- | --- | --- | --- | --- | --- |
|  | (n) | (%) | (n) | (n) | Total | After fumagillin treatment5 |
| Southern Alberta | 117 | 27 | 85 | 47 | 50 | 40 |
| Control | 30 | 30 | 21 | 13 | 11 | 8 |
| Fumagillin | 29 | 24 | 22 | 15 | 12 | 9 |
| Protein | 28 | 29 | 20 | 9 | 12 | 10 |
| Fumagillin & Protein | 30 | 27 | 22 | 10 | 15 | 13 |
| Northern Alberta | 123 | 47 | 65 | 14 | 92 | 36 |
| Control | 31 | 39 | 19 | 5 | 21 | 10 |
| Fumagillin | 30 | 30 | 21 | 5 | 28 | 12 |
| Protein | 31 | 68 | 10 | 1 | 21 | 7 |
| Fumagillin & Protein | 31 | 52 | 15 | 3 | 22 | 7 |
| Prince Edward Island2 | 110 | 85 | 13 | 3 | 37 | 32 |
| Control | 24 | 96 | 1 | 0 | 8 | 8 |
| Fumagillin | 28 | 79 | 6 | 2 | 9 | 9 |
| Protein | 26 | 88 | 3 | 1 | 11 | 8 |
| Fumagillin & Protein | 32 | 91 | 3 | 0 | 9 | 7 |

1 Data shown reflect colony assignment to treatment group. Some colony deaths occurred before the first fumagillin application, and thus do not reflect the effectiveness of that treatment.

2 Data for PEI includes original colonies and splits made in 2014 but not the 12 additional splits produced in August 2015.

3 At the May 2016 inspection date.

4 Includes both natural and beekeeper-initiated queen replacements. Excludes queen failures associated with colony death. Some colonies experienced multiple queen events. These numbers represent all known queen replacements.

5 The first fumagillin application did not occur until September 2014. Queen deaths after fumagillin treatment are shown separately because fumagillin has previously been recommended for preventing queen loss [27].
