# Supplementary material for: The effects of protein supplementation, fumagillin treatment, and colony management on the productivity and long-term survival of honey bee (Apis mellifera) colonies
Source: PLoS One. 2024 Mar 15;19(3):e0288953. doi: 10.1371/journal.pone.0288953 (PMC10942092; doi:10.1371/journal.pone.0288953)
Supplement: Supplementary file 18 [file pone.0288953.s022.odt]

S10 Table. Characteristics of split and non-split colonies at PEI.

| Year | Colony status | Viable colonies | Winter survival rate | Mean fall cluster size | Mean fall colony weight |
| --- | --- | --- | --- | --- | --- |
|  |  | Aug | Nov | May |  |  |  |
|  |  | (n) | (n) | (n) | (%) | Inter-frame spaces | (kg) |
| 2014 | Not Split | 42 | 33 | 31 | 94% | 7.6 | 43 |
|  | Split - Parent Colony1 | 34 | 33 | 26 | 76% | 7.3 | 37 |
|  | Split - Daughter Colony | 34 | 30 | 17 | 57% | 4.0 | 34 |
| 2015 | Not Split | 28 | 28 | 9 | 32% | 6.3 | 58 |
|  | Split - Parent Colony | 10 | 10 | 4 | 40% | 5.5 | 56 |
|  | Split - Daughter Colony | 12 | 12 | 5 | 42% | 4.3 | 49 |

1 The parent is the colony that retained the original box and queen after the split.
