# Supplementary material for: The effects of protein supplementation, fumagillin treatment, and colony management on the productivity and long-term survival of honey bee (Apis mellifera) colonies
Source: PLoS One. 2024 Mar 15;19(3):e0288953. doi: 10.1371/journal.pone.0288953 (PMC10942092; doi:10.1371/journal.pone.0288953)
Supplement: Supplementary file 19 [file pone.0288953.s023.odt]

S11 Table. Weights of honey bee colonies before and after winter.

| Region | Winter | Fall weight1 | Spring weight | Average feed consumption |
| --- | --- | --- | --- | --- |
|  |  | kg | kg | kg/winter | g/day |
| SAB2 | 2014-15 | 56.7 ± 0.73 | 40.6 ± 0.6 | 16.1 | 109 |
| SAB | 2015-16 | 56.6 ± 0.4 | 38.3 ± 0.5 | 18.3 | 114 |
| NAB | 2014-15 | 56.4 ± 0.5 | 38.1 ± 0.5 | 18.3 | 108 |
| NAB | 2015-16 | 64.5 ± 0.7 | 40.7 ± 0.6 | 23.8 | 123 |
| PEI | 2014-15 | 39.6 ± 0.8 | 25.7 ± 0.5 | 13.9 | 86 |
| PEI | 2015-16 | 57.7 ± 2.6 | 37.6 ± 2.1 | 20.1 | 137 |

1 Fall and spring data both exclude colonies that were not viable at the April weight measurement. Weight was measured as the sum of the weights of the upper and lower brood boxes, with the bees present in the box.

2 Abbreviations: SAB: Southern Alberta; NAB: Northern Alberta; PEI Prince Edward Island.

3 Mean ± SE
