# Supplementary material for: The effects of protein supplementation, fumagillin treatment, and colony management on the productivity and long-term survival of honey bee (Apis mellifera) colonies
Source: PLoS One. 2024 Mar 15;19(3):e0288953. doi: 10.1371/journal.pone.0288953 (PMC10942092; doi:10.1371/journal.pone.0288953)
Supplement: Attachment — Submitted filename: Response to Reviewers.doc [file pone.0288953.s024.doc]

Dear Dr. Rueppell or Editor, PLOS ONE

Thank you for considering our paper,

PONE-D-23-21321

The effects of protein supplementation, fumagillin treatment, and colony management on the productivity and long-term survival of honey bee (*Apis mellifera*) colonies.

Note that we have made a minor change to the title by dropping the words "in Canada".

Below, as requested, are our responses to comments raised by the reviewers and yourself.

Yours truly,

Michael Peirson

Editor Comments:

1. Please ensure that your manuscript meets PLOS ONE's style requirements, including those for file naming.

We have adjusted the supplemental file names, as indicated.

2. Please remove any funding-related text from the manuscript.

Done.

3. We are unable to open your Supporting Information file "Supplement 4 Statistical Analysis.Rmd". Please kindly revise as necessary and re-upload.

S4_File has been included only for transparency in statistical methods and is not needed to understand the work. It contains the R code used in the analysis; the output of the analysis (more likely of interest to readers) is provided as S5_File.html.

The file was created with Rstudio, and there is no defect in it as submitted; we have checked, and it opens and runs correctly. To run the program, the datafiles (S2_File and S3_File) should first be saved to the same location as S4_File. I have added detail about the requirement for RStudio and R to the file caption.

Additional Editor Comments:
The complex study design is more a weakness than a strength, despite the large sample size. I also agree with the reviewers that the comparison among different regions is difficult (due to many co-occurring differences that cannot be disentangled) and therefore should be minimized, in contrast to evaluation of supplemental feeding and Nosema treatments, which are valuable. Furthermore, I agree with both reviewers that the manuscript needs to become more accessible to readers by streamlining and shortening its presentation considerably, including the modeling. For methods and results, this means reorganizing and transferring some material to supplemental files. For the discussion, it means focusing on the meaningful messages and issues, as suggested by both reviewers.

We have made efforts to reorganize and substantially streamline the length of the manuscript. As requested, most of the text of the methods section, along with the tables and half the figures from the results section, have been transferred to supplemental files. The text of the Results and Discussion has been edited and the overall word count has been reduced from more than 12,000 to 9,200.

We agree that the design is complex and that much of this complexity is related to differences in management specific to regions, particularly in PEI. To make the manuscript more accessible, we have made efforts to highlight the comparisons related to treatments, and reduce those among regions. We recognize that uniform colony management practices across the three sites would have certainly made our analysis much simpler and the results more straightforward to describe. Nevertheless, we made a conscious decision to study three “real-world” beekeeping management styles in order to obtain results that would be broadly applicable and relevant to commercial beekeeping in Canada.

Reviewer #1: General comments:

This study aimed at assessing the effect of supplemental protein feeding and antibiotic treatment against nosema disease on the survival, development and productivity of honey bee colonies. The study was conducted over a two-year period in three different regions of Canada. The results were inconsistent across regions and seasons. In some regions treatments had a positive effect whereas in others, the same treatments (particularly supplemental feeding) had a negative effect on the variables measured. These results are expected because there were many uncontrolled variables that affected the performance of the colonies, like differences in geographic location, climate, colony management, time of year when the colonies were established, colonies that were divided once versus twice a year, etc. The value of the study is that it used many colonies (>350) and that it was conducted for two years (usually these type of studies are conducted for only one year). However, the authors should consider toning down their conclusions and warning the reader that these results (particularly those related to the negative effects of protein supplementation) should be taken cautiously and that future studies should be planned to try to standardize experimental conditions in a way that experimental error can be minimized.

Reviewer One's summary is accurate but we do not feel that our conclusions are stated in a manner inconsistent with the limitations of our design. As the second reviewer recognized, our principal conclusion is that the effects of protein and fumagillin depend on local conditions. Based on broad-scale knowledge of beekeeping practices in North America, this finding is hardly surprising. We were surprised by the negative long-term effect of protein supplementation. Nevertheless, we have been careful not to extend our conclusions beyond what we felt our data showed: long-term use of protein supplements was either ineffective or even potentially counterproductive. This result is consistent with other research examining supplemental feeding where even from short term studies have shown that it is not equivalent to natural forage, and occasionally may have negative consequences. This point is also included in the Discussion.

The aspects of the study that the reviewer refers to as uncontrolled elements or experimental error, arise from the differences among regions and beekeepers. Such differences exist throughout the beekeeping industry and the choice to capture them in some degree was deliberate. Within region, methods and results were controlled as much as possible and colonies managed consistently. The one exception to this was colony splitting in PEI (done at the discretion of the beekeeper). Indeed, we could have answered this objection by parsing our results among separate publications, or by dropping PEI entirely. In fact, we spent considerable time in evaluating whether PEI should be dropped from this paper, and also worked through the statistical analysis on a within-region basis (not shown). With this rigorous examination of the data we learned that the conclusions would not have been different, with the exception of loss of statistical power. Keeping PEI, though more complex, also highlighted the challenges faced by maritime beekeepers and allowed for full transparency in the data we collected.

Specific comments:

1. For future submissions please number lines and pages for easier review.

Corrected.

2. Introduction, page 11. Please italicize Varimorpha (scientific name).

Corrected, at lines 84-92 of the revised document.

3. Methods, page 13. It is not clear from the text if the colonies of all three regions used the same queen source (Kona queens). Please clarify.

Methods details have been moved to S1_File. The text (at line 9 of S1_File) has been adjusted to read:

*In each region, newly mated Carniolan queens (Kona Queen Company, HI, USA) were introduced to each colony established at the beginning of the study.*

4. Methods, page 14. It is not clear how PEI beekeepers supplemented their colonies. Please elaborate on the matter.

We are uncertain what information the reviewer is asking for here. If the reviewer is referring to the ordinary feeding practices of the beekeeper in PEI, we believe this beekeeper did not ordinarily provide protein supplements, as described at lines 122-125 (somewhat revised):

*Beekeepers applied their normal early spring and fall feeding practices, which for Alberta producers, but not PEI, included providing commercial protein supplements (Global Patties with 15% pollen; Global Patties, Airdrie, AB, Canada) to all colonies in spring.*

If the reviewer is referring to the quantity of additional protein supplement applied at PEI, the information we have is in Table D of S1_File. This beekeeper did not provide a detailed report of quantities fed to us. We believe that consumption rates in PEI were approximately 1/2 patty every two weeks.

5. Results and discussion. Winter colony mortality was higher in Northern Alberta (54%) and colonies were less populated. The authors attribute those results to the effect of protein supplementation. However, it is not well explained why supplemental feeding would be detrimental to populations and colony survival. It is important to hypothesize as to why this could be. Additionally, please discuss that rather than the effect of the supplemental treatment, these detrimental effects could have been caused by V. destructor infestations. There is plenty of evidence that mite infestations are more frequently associated with colony losses during winter than any other cause. If possible, show data of mite levels for all regions and seasons, which could shed light on the results and conclusions of this study.

It is imprecise to say that we *attribute* the losses in Northern Alberta to protein supplements, because the finding is an observed result of this controlled trial, not an interpretation. We state (at line 104 and again at line 540) that the effect of protein was expected to be positive. As an unexpected finding, the contrary result would be important to report even if we had no hypotheses that might explain it. However, the following hypotheses are provided:

Lines 562-572 describe evidence that protein supplements may be associated with shorter average adult bee lifespans.

Lines 572-583 give a principal hypothesis for how protein could lead to shorter lifespans (increased nosema prevalence).

Lines 585-598 give four hypotheses as to why protein fed as supplements might fail to produce the benefits expected from naturally-foraged pollen.

The reviewer's request for additional information on *Varroa destructor* is reasonable. We have added a brief summary at lines 264-266:

*Varroa destructor levels were low and well managed throughout the experiment, and were not biased among treatment groups. Mites per hundred bees averaged over all dates were 0.02 (SAB), 0.08 (NAB), and 1.7 (PEI).*

We have deliberately limited ourselves to effects arising from the design elements of the experiment: protein supplements, fumagillin, and region. However we also collected a very great deal of information related to pesticides, pests, and diseases, which we hope to publish in a series of subsequent papers. A draft paper expected to cover our observations of Varroa, visible diseases, and queen supersedure in some detail is currently in preparation.

6. Page 40. Consider using another word or deleting the word “dramatically” related to honey production. Not scientific jargon. Was it significant? If so, please use that word.

Done (Line 527).

7. Page 45. Colonies accepted less feed with fumagillin contrary to what occurs in cage experiments. Why is that? In cage experiments bees fed fumagillin tend to consume more syrup. Please try to explain this discrepancy.

While we are aware that certain pesticides are believed to increase the attractiveness of nectar or syrup, we have never heard of this for fumagillin. Indeed, we have the impression that the problem with feed acceptance which we report is already well known among commercial beekeepers, though seemingly not in the literature. The following publication describes a cage study in which fumagillin had no effect on consumption, which is contrary to the reviewers assertion:

Webster, T. C. (1994). Fumagillin affects *Nosema apis* and honey bees (Hymononopterai Apidae). J. Econ. Entomol. 87(3): 601-604.

8. Page 47. “Poor nutrition kill hives.” Hives are made from wood or other materials and are not live organisms. Do you mean colonies? If so, please reword.

This change has been made.

9. A very high proportion of the original queens were lost or superseded. The period of queen replacement has a direct impact on colony populations. Does queen supersedure had to do with results on colony populations? Please discuss.

In short, yes. As mentioned in the response to Comment 5, the effects of queen supersedure, varroa, and visible disease on colony survival, size and productivity are intended to be addressed in a second paper, now being drafted. As the reviewer points out, the current paper is exceedingly long and we have chosen to examine other data collected in subsequent contributions.

10. Tables. Please reduce the font size of subscript legends.

Tables have been moved to supplementary materials, and legend text size has been reduced.

11. Table 3. The weight difference results from differences in feed and bee populations, not only feed weight. Please mention that.

The reviewer’s comment is correct and has been added to the footnote of Table S11.

12. Figures. Please use different colours for bars representing different treatments. The same colours are confusing.

Colour has been used throughout to designate region: green for SAB, red for NAB, blue for PEI, goldenrod for results combined across regions. The treatments are indicated with crosshatching. In examining the use of additional colour patterns to the figures, we felt this was potentially more confusing to a naïve reader.

13. Authors should consider limiting the paper to show results of the region or regions that had experimental colonies with more uniform and consistent management. For example, only Northern or Southern Alberta or both. That would reduce the size of the paper and will also provide data from colonies that were less affected by the uncontrolled variables. The conclusions would be more reliable in my opinion.

This point is not unreasonable; indeed it is so reasonable that we have already done so at the analysis stage. Results were modeled within each region, and then for Alberta colonies only, before settling on models that expressed the full complexity of the experiment. The conclusions of a paper that excluded PEI, or that modeled effects of treatments on a within-region basis only would have been largely the same as those presented, but there would have been a loss of power.

14. Please shorten the paper by at least one third. It is unusual to have manuscripts of more than 45 pages. Each section of the paper could be shortened. There are too many figures too (12). Consider presenting only the most representative data in figures and tables. If necessary, present some of the data in supplemental tables and/or figures to reduce the number of tables and figures in the manuscript.

Though difficult, we have made considerable efforts to do so. The manuscript is now considerably shorter in length.

Reviewer #2: This study examines the effects of two treatments (fall Fumagillin B treatments against Variamorpha spp. (Nosema spp.) and continuous pollen patty supplements) on long term productivity and colony survival in Canadian commercial colonies. Both treatments are applied prophylactically to reduce disease incidence and improve colony productivity and survival in colonies in many regions. The authors found that fumagillin treatments and pollen patty treatments have little to negative long term effects on colony productivity and survival, despite their reputation to the contrary among beekeepers. The study follows and evaluates these colonies very thoroughly and extend well beyond the period usually covered by similar studies. These findings are important to relate because they demonstrate neutral to negative effects of perhaps the two most recommended colony treatments aside from Varroa mite treatments. Despite major regional and management differences, an absence of benefits is observed for both treatments across all three regions. Studies such as this that report neutral to negative effects of common treatments are essential to public discussion of their value and should be reported.

Thank you for the accurate summary. The results from fumagillin, aside from feed acceptance, were generally positive despite being inconsistent. We appreciate this reviewer valuing our efforts to collect data across all three regions.

The main problems of the study stem from exactly what the authors attempt to capture, namely, treatment effects with regional differences in management practices. Not surprisingly, the authors report that regional differences swamp treatment effects of fumagillin and pollen protein supplements. One real problem is that the different regional practices regarding colony establishment (packages, splits, nucs) start the colonies with very different sized populations and compositions, which in a sense make head on comparisons with performance data somewhat tenuous (apples to oranges comparisons). Unfortunately the observed regional differences could be attributed to different colony establishment practices as well as environmental differences. The study is particularly confounded by the late summer splits performed by the PEI beekeeper on what seem to be colony strength based criteria. This beekeeper action is understandable to anyone who has worked on longitudinal studies with commercial beekeepers (sometimes some of them do things that work against the experimental design). However, the claims that colony size could be predicted by adding original and daughter split colonies together are somewhat of a stretch. Problems notwithstanding, what is striking though is that a lack of positive benefits were observed in three quite different colony environments.

We appreciate the sympathetic comment about experiments involving commercial beekeepers; all too true! We also appreciate the final sentence of the paragraph; indeed, this is the point of presenting all three regions at once rather than writing three smaller papers.

The manuscript itself is very extensive in presentation of modeling and modeling immediate results but light on discussion of these results to relevant literature. The modeling results are extensive framed and discussed in detail with thoughtful explanations of effect causes, but need to be streamlined to avoid overwhelming the paper. The number of figures (12) are quite high and some of them could be converted into supplemental figures to streamline the paper. The authors discuss their findings nicely with the relevant literature but may want to expand comparisons based on timing and duration of treatments (especially pollen patty substitution). The overall message that treatment efficacy varies regionally with environmental and regional practices is an eminently sensible one and should be the basis of whether such practices are locally useful for their intended purposes.

As described above, we have greatly reduced the length of the text, and half the figures have been moved to the supplemental materials. Hopefully this makes the work more readable. We have made some minor changes to the text around lines 532-540 to emphasize that the prior studies we reference were short term studies showing benefits in spring.

Specific comments

(general) The methods, results, and explanation of statistical approaches are very detailed and professional here. The authors handled a considerable amount of statistical comparisons with nuance and appropriate explanation.

Thank you.

(general, p47 1st sentence on protein supplement being above and beyond normal, continuous). The continuous protein supplementation is a critical aspect of the study that differentiates it from other pollen substitution studies. The authors emphasize this in the Abstract and in the Discussion, but this should be also be noted in the experiment outline at the end of the Introduction. Likewise, comparisons with other studies need to take into account the treatment timing and duration of those studies. The authors did a fairly decent job of this but it is worth going through the drive the distinction home.

We have added the word "continuously" at line 98, and some detail about references 50 (Matilla and Otis) and 18 (Hoover) at line 536.

(general) The authors need to be careful about presentation of non-significant trends and tendencies. Several times in the manuscript differences are presented before non-significant test results are presented. One suggestion would be to note quickly the absence of a significant difference but then discuss trends. I should note that this is simply a difference of presentation style and that the authors have been thorough and forthright in presentation of their results.

This has been changed in a number of places, particularly the section describing colony and queen survival (lines 209-257).

(general) Consider using the term “surviving colonies” to describe statistics gathered from surviving colonies only through the manuscript. Removal of data from colonies that later perished in the study is understandable but may lead to different results that if this data was left in at time points when these colonies were alive.

The term "surviving colonies" has been used in several places; for example at line 234.

The reviewer's description here may not perfectly reflect what we did, which is described at lines 181-186. When a colony does not have a laying queen and sealed worker brood, it can be impossible to determine whether it is a permanently queenless colony, a queenright but temporarily broodless colony, or a colony undergoing supersedure. We wanted to include superseding and temporarily broodless colonies in the dataset because these are normal conditions in the life of the colony. We did not want to include dying colonies in the dataset because the characteristics of dying colonies are driven by the fact that they are dying, not by colony management. For example, consider a colony that appeared to be queenless in May 2015, was not subsequently found to be queenright, and had no remaining bees in October 2015. Measurements of that colony from May, June, and August 2015 will be in the datafile, but have been excluded from the summary statistics and analysis. Measurements for April 2015 and earlier remain in the dataset and are analyzed.

general, p49-50) Blueberries have a mixed reputation in their impacts on pollinating honey bee colonies. In some regions, impacts appear on honey bee colonies appear to be severe. Any comments on colony survival in blueberry pollinating sites here, i.e. do the colonies often have trouble or is there sufficient forage to make it through?

Good question. We have added text at lines 632-639:

*We did not find evidence that colonies of the blueberry pollinator suffered from inadequate forage. The very high rate of brood production of both supplemented and non-supplemented colonies in PEI, particularly in 2014, indicates that forage was not limiting in the region. In addition, if pollen sources during blueberry pollination had been inadequate, protein-supplements should have produced an increase in sealed brood cell count in June 2015. No such effect was found. Nor did protein supplements affect the amount of pollen collected by colonies in blueberries (Fig S7, PEI before July 1).*

p20 pollen collection p42 Fig 10. The methods and heading describes how pollen was trapped for canola pollinating colonies in 2014. Can you add details for the PEI colonies?

Unfortunately no pollen was collected in 2014 in PEI because of the late start date there, and a lack of staff and equipment.

P23 Cluster size and colony weight models 2nd sentence The approach of dropping colonies that died during overwintering from the fall analysis is conservative but by choice exclude colonies in decline. How do the November comparisons compare if these doomed colonies are included?

We have not altered the text of the paper in answer to this question. The decision to drop colonies that died before April from the November analysis was made relatively early in the model optimization process to ensure that we were comparing apples to apples. In response to the question, I did re-run the cluster size model from the paper on a dataset that included all colonies in November; results were similar.

p23 Results Colony survival 3rd sentence “104 of 122” The count of PEI colony failures appears to be off.

The 12 daughter colonies produced by splitting in 2015 have been excluded from Table S9 (previously Table 1) because the time period during which they could have died or lost their queen was so much shorter than all the other colonies. This is indicated in footnote 2 of the table. However, they are included in the summary in the text (line 212).

p27 Table 1 d count of queen replacements Not clear here on whether multiple replacements in a colony would be counted once or separately in these tallies.

These were counted separately. Changed the text of footnote 4 to: "These numbers represent all known queen replacements."; perhaps this change clarifies the situation.

p27 “…occurred during the winters (…, than during the summers …” Can you consider use of other terms to describe these halves of the year? Cold season and warm season come to mind (but are probably too awkward). Winters and summers seems to leave out fall and spring builds.

The summary of losses by season has been dropped in response to the request that we make the paper shorter and more focused on treatment effects.

p28 Queen survival 4th sentence. Perhaps substitute “A lower proportion of colonies in the protein supplemented groups" for “Fewer colonies” given the disparity in colony survival.

The entire paragraph has been re-written (lines 233-242), in part with the intent of clarifying that comparisons of original queen survival rates are made among the surviving colonies, not among total colonies, which I take to be the point of the reviewer's suggestion.

Fig2 and Fig3 headings. For the PEI colonies, are the adult and brood population counts only from the original split colonies or both daughter and original colonies given the differences in bee splits here?

These figures are now designated S2 & S3 Figs in response to the general request to shorten the paper. All measurements from viable colonies are shown as-is. Daughter colonies were not excluded nor their populations attributed to the parent. However at the August 2014 inspection in PEI, none of the daughter colonies had sealed brood. Rather than include a large number of zeros, which would have given an average result that didn't describe either the parents or the daughters, we included only data from the parent colonies. The caption for S3 Fig has been adjusted to reflect this.

p33 The splits were a beekeeper decision … p34 Thus, it is probable that starvation … This is a realistic problem in longitudinal studies and well presented here.

Thank you.

p36 and p38 PEI colony weights and starvation Table 3. The high colony masses of the PEI colonies are quite interesting in light of the likely starvation of some of these colonies. The 2015-2016 colonies appear to be of near average mass of the SAB and NAB colonies. Did the PEI beekeepers attempt any supplemental sugar feeding as part of their overwintering management?

The beekeeper did not inform us of any changes to his management plan, but the reviewer is correct, he achieved much better results, with respect to colony weight, in the second winter.

p44 Discussion 1st paragraph. The discussion of how regional differences were much stronger than treatment differences is illustrated quite well here. It is an honest point.

Thank you.
