# Supplementary material for: The effects of protein supplementation, fumagillin treatment, and colony management on the productivity and long-term survival of honey bee (Apis mellifera) colonies
Source: PLoS One. 2024 Mar 15;19(3):e0288953. doi: 10.1371/journal.pone.0288953 (PMC10942092; doi:10.1371/journal.pone.0288953)
Supplement: Attachment — Submitted filename: Response to reviewers second round.odt [file pone.0288953.s025.odt]

Dear Dr. Rueppell or Editor, PLOS ONE 

Thank you for considering our paper,

PONE-D-23-21321
The effects of protein supplementation, fumagillin treatment, and colony management on the productivity and long-term survival of honey bee (Apis mellifera) colonies.

Below, as requested, are our responses to comments raised by the reviewers and yourself.


Yours truly,


Michael Peirson


Please review your reference list to ensure that it is complete and correct. If you have cited papers that have been retracted, please include the rationale for doing so in the manuscript text, or remove these references and replace them with relevant current references. Any changes to the reference list should be mentioned in the rebuttal letter that accompanies your revised manuscript. If you need to cite a retracted article, indicate the article’s retracted status in the References list and also include a citation and full reference for the retraction notice.

I have reviewed the reference list and it appears to be correct and complete. 
The citation at line 178 has been corrected from [35] to [37].
The citations at line 194 have been corrected from [38, 39] to [40, 42]

Reviewer comments:

Reviewer #1: Thanks for addressing my concerns. The paper is much better now. Still a little bit long but I appreciate that the authors had made an effort to shorten this complex report

We are glad to hear this.

Reviewer #2: The authors have substantially revised their manuscript so that it is more compact, concise, and structurally easy to interpret. As noted by the Editor and reviewers, the main featured aspects of this study (regional comparisons, differences in protocols and local practices) were also highly problematic. At worst, the “telescoping” of effects from regional differences, beekeeper practices, and protocol differences could lead to overstatement of treatment effects. More so than studies of less substantial design, the manuscript needed to be trimmed, focused, and tightly structured in its presentation.

The revised manuscript achieves this balance. The authors refocused on treatment differences but also critically acknowledged key regional and beekeeper practices differences. The authors compartmentalized the presentation of the results in such a way that readers would not become overwhelmed or lose track of results. The refocus on treatment effects in the context of regional differences adequately wrangles an information dense presentation into its component sections. The number of figures, tables, and supporting information has been reduced both in the manuscript and by shunting large parts to the Supplemental Information sections. At the same time, the authors show openness and nuance in consideration of their design and results. They do not shy away from problems or flaws that arose during the experiment. The emphasis on reduced fumagillin treatment syrup intake as a possible stress factor (and it’s occurrence primarily in one region) is an area for management improvement.

We are pleased to see this positive response.

I would suggest that the authors revisit their Figure and Supplemental Information citations carefully. Fig 1 and S1 Fig are missing from the reviewer’s copy of the manuscript but the corresponding results appear to be in line with data given in the Supplemental Information figures and files.

I am surprised that the reviewer's copy did not include Fig 1 and S1 Fig. 
On checking the online submission site, it appears that Fig 1 was left out of the revised submission. This will be corrected with the current submission. Fig_S1 was included with the previous submission, and no change has been made.

The manuscript references figures and supplements as follows:

Line 119 S1 File and S1 Table. General Methods. Appear to be correctly cited.
Line 131 S2 & S3 Table. Protein treatment group assignments and quantities fed. Correctly cited.
Line 140 S2 Table. Fumagillin treatment group assignments. Correctly cited.
Line 141 S4 Table. Timing and method of fumagillin treatments. Correctly cited.
Line 151 S5 Table. Colony assessment dates and types. Correctly cited.
Line 157 S1 File. Population assessment methods. Correctly cited.
Line 157 S1 Fig. Illustration of population assessments. Correctly cited.
Line 157 S5 -S8 Tables. Dates and calculations related to population assessments. Correctly cited.
Line 162 S1 File. Population assessment methods. Correctly cited. 
Line 190 S1 File. Details of statistical methods. Correctly cited.
Line 191 S2-S5 File. Project datasets and statistical analysis. Correctly cited.
Line 212 Fig 1. Correctly cited
Line 212 S9 Table. Colony and queen survival to the end of the experiment. Correctly cited.
Line 214 S10 Table. Effect of splitting on survival, cluster size, and colony weight.
Lines 226-228 Fig 1. Correctly cited
Line 235 S9 Table. Queen survival. Correctly cited.
Line 244. S9 Table. Queen survival. Correctly cited.
Line 264 S5 File. Statistical summary. Correctly cited.
Line 270 S2 Fig. Adult bee counts by region, date, and treatment group. Correctly cited.
Line 271 S3 Fig. Sealed brood counts by region, date, and treatment group. Correctly cited.
Line 274. Incorrectly cited. Should be S4 Fig. Cluster size.
Line 282. Incorrectly cited. Should be S2-S4 Figs.
Line 318 S2 Fig. Adult bee counts. Correctly cited.
Line 323 Fig 2. Correctly cited
Line 347 Fig 2. Correctly cited
Line 375 Fig 3. Correctly cited
Line 377 Fig 3. Correctly cited
Line 401 S11 Table. Colony weights in winter. Correctly cited.
Line 404 S10 Table Effect of splitting on colony weight. Correctly cited.
Line 407 Fig 4. Correctly cited
Line 409 S5 Fig. Effect of fumagillin on weight. Correctly cited.
Line 430 Fig 5. Correctly cited
Line 445 S6 Fig. Trapped Pollen. Correctly cited.
Line 451 S7 Fig. Trapped Pollen 2015. Correctly cited.
Line 461 Fig 6. Correctly cited.
Line 638 S7 Fig. Trapped Pollen 2015. Correctly cited.


line 248 Are there stats to go with the Cox hazards on surviving queens?

Added the following at line 248:
(hazard ratio for protein: 1.1; for fumagillin: 0.9; p>0.05 in both cases).


line 264 The authors may want to slightly rephrase the section on mite infestation rates to support later statements on PEI colony disease/parasite stressors (paragraph starting on 618, specifically 629). This reviewer agrees that the average mite counts for PEI colonies (1.7 mites/100 bees) are marginally high (approaching treatment thresholds, which of course vary by region, local practices and time of year) and may be a cause for concern under the right conditions as noted in the Discussion.
Changed text to:
Varroa destructor levels were well managed and not biased among treatment groups. Mites per hundred bees averaged over all dates were 0.02 (SAB), 0.08 (NAB), and 1.7 (PEI).

line 273 Weren’t cluster sizes reported in S4 Fig not S3 Fig?
The reviewer is correct, and this has been changed.

line 281 Should the supporting figures be S2 Fig, S3 Fig, and S4 Fig rather than S1 Fig, S2 Fig, and S3 Fig given the topic covered?
The reviewer is correct and this has been changed.

line 298 Marginally non-significant (p=0.07)?

Okay. Phrasing changed from "marginally significant" to "marginally non-significant"


line 560 An interesting feature of this study is that the pollen supplementation treatments occur in excess of local beekeeper practices (both forage and pollen supplementation) that presumably provide minimal nutrition in most years. One minor point – the pollen supply and pre-existing beekeeper supplementation practices are probably adequate to allow beekeepers to produce in these areas.
changed to read as follows:
in regions with a successful bee industry the pollen supply and existing feeding practices are probably adequate


574 Authors may want to examine studies detailing increased Vairimorpha spore counts in hindgut in pollen fed bees for their companion paper. The idea that pollen feeding in supplements may temporarily directly benefit the feeding adult but not future generations is interesting, particularly if such supplementation leads to higher disease spore exposures.
Thank you for the suggestion.
